# Arabidopsis histone deacetylase HD2A and HD2B regulate seed dormancy by repressing DELAY OF GERMINATION 1

**DOI:** 10.3389/fpls.2023.1124899

**Published:** 2023-05-29

**Authors:** Yongtao Han, Elisabeth Georgii, Santiago Priego-Cubero, Christoph J. Wurm, Patrick Hüther, Gregor Huber, Robert Koller, Claude Becker, Jörg Durner, Christian Lindermayr

**Affiliations:** ^1^ Institute of Biochemical Plant Pathology, Helmholtz Zentrum München, German Research Center for Environmental Health, München, Germany; ^2^ Genetics, LMU Biocenter, Ludwig-Maximilians-Universität München, München, Germany; ^3^ Institute of Bio- and Geosciences, IBG-2: Plant Sciences, Forschungszentrum Jülich GmbH, Jülich, Germany; ^4^ Chair of Biochemical Plant Pathology, Technische Universität München, Freising, Germany; ^5^ Institute of Lung Health and Immunity, Comprehensive Pneumology Center, Helmholtz Zentrum München, Member of the German Center for Lung Research, München, Germany

**Keywords:** *Arabidopsis thaliana*, DELAY OF GERMINATION 1, histone acetylation, plant-specific histone deacetylases, seed dormancy, seed germination

## Abstract

Seed dormancy is a crucial developmental transition that affects the adaption and survival of plants. Arabidopsis DELAY OF GERMINATION 1 (DOG1) is known as a master regulator of seed dormancy. However, although several upstream factors of DOG1 have been reported, the exact regulation of DOG1 is not fully understood. Histone acetylation is an important regulatory layer, controlled by histone acetyltransferases and histone deacetylases. Histone acetylation strongly correlates with transcriptionally active chromatin, whereas heterochromatin is generally characterized by hypoacetylated histones. Here we describe that loss of function of two plant-specific histone deacetylases, HD2A and HD2B, resulted in enhanced seed dormancy in Arabidopsis. Interestingly, the silencing of *HD2A* and *HD2B* caused hyperacetylation of the *DOG1* locus and promoted the expression of *DOG1* during seed maturation and imbibition. Knockout of *DOG1* could rescue the seed dormancy and partly rescue the disturbed development phenotype of *hd2ahd2b*. Transcriptomic analysis of the *hd2ahd2b* line shows that many genes involved in seed development were impaired. Moreover, we demonstrated that HSI2 and HSL1 interact with HD2A and HD2B. In sum, these results suggest that HSI2 and HSL1 might recruit HD2A and HD2B to *DOG1* to negatively regulate *DOG1* expression and to reduce seed dormancy, consequently, affecting seed development during seed maturation and promoting seed germination during imbibition.

## Introduction

As the initial phase of a plant’s life cycle, seed germination is essential for seedlings’ establishment and growth. The proper timing of seed germination ensures plant development under suitable conditions and is determined by seed dormancy release. Seed dormancy is an evolutionary adaptive mechanism that can be simply defined as viable seeds that fail to germinate under favorable conditions (Finch-Savage and Leubner-Metzger, 2006). Dormancy is imposed by phytohormones and genetic factors, established during seed maturation, persists in mature seeds, and can be released by after-ripening and seed stratification (Gubler et al., 2005). Abscisic acid (ABA) and gibberellin acid (GA) are recognized as essential endogenous phytohormones that play antagonistic roles in regulating seed dormancy. DELAY OF GERMINATION 1 (DOG1; At5g45830) was identified as a master regulator of primary dormancy in a QTL analysis for seed dormancy using a set of recombinant inbred lines derived from a cross between low dormant accession Landsberg erecta (Ler-0) and very dormant accession Cape Verde Islands (Cvi-0) (Alonso-Blanco et al., 2003; Bentsink et al., 2006). *DOG1* encodes a nuclear protein with unknown biochemical function and is mainly expressed in seed (Bentsink et al., 2006; Nakabayashi et al., 2012). The DOG1 protein accumulates during seed maturation and peaks in freshly harvested seeds. At this developmental stage, the DOG1 level determines the seed dormancy level (Nakabayashi et al., 2012). Although the DOG1 protein persists during dry storage and seed imbibition, the after-ripened seeds lose their dormancy. This indicates a loss of DOG1 activity at the after-ripening stage, which might be caused by an altered protein structure (Nakabayashi et al., 2012). Recent evidence suggests that multiple factors are involved in regulating *DOG1* expression. Nakabayashi et al. (2012) found that lower seed maturation temperature upregulated *DOG1* expression and increased seed dormancy. This might be triggered by increased expression of transcription factor (TF) bZIP67 which can bind to the *DOG1* promoter (Bryant et al., 2019). Additionally, *DOG1* expression is regulated by epigenetic regulators. Histone demethylases LDL1/LDL2 and histone methyltransferases KRYPTONITE (KYP)/SUVH4/SUVH5 repress *DOG1* during seed maturation (Zheng et al., 2012; Zhao et al., 2015). *DOG1* expression also can be regulated by alternative splicing, cis-acting antisense noncoding transcript (as*DOG1*), and histone acetylation (Nakabayashi et al., 2015; Fedak et al., 2016).

B3 domain-containing transcriptional repressors HIGH-LEVEL EXPRESSION OF SUGAR INDUCIBLE2 (HSI2) and HSI2-LIKE1 (HSL1) play also critical roles during plant reproduction and seed germination (Qüesta et al., 2016; Schneider et al., 2016; Yuan et al., 2021). HSI2 and HSL1 can form dimers to bind on the *DOG1* promoter recruiting components of polycomb-group proteins for consequent deposition of H3K27me3 marks resulting in repression of *DOG1* (Li et al., 2019). Additionally, HSI2 and HSL1 interact with histone deacetylase HDA6 and HDA19 and participate in down-regulating seed maturation gene expression in Arabidopsis seedlings (Zhou et al., 2013; Chhun et al., 2016).

HDAs are enzymes that catalyze the deacetylation of histone and non-histone proteins (Zhao et al., 2010). Histone deacetylation leads to chromatin compaction, which is usually transcriptionally inactive. Arabidopsis has 18 HDAs, which are grouped into 3 subfamilies type I RPD3-like HDAs, HD-tuins, and sirtuins. The RPD3-like HDAs have a conserved HDA domain that shares high homology with the yeast transcriptional regulator RPD3 (reduced potassium deficiency 3). HD-tuins (HD2-type HDAs) are plant-specific and contain 4 members, HD2A, HD2B, HD2C, and HD2D. These proteins are related to the FKBP family of *cis-trans* peptidyl-propyl isomerases (Aravind and Koonin, 1998; Dangl et al., 2001). Although inhibition or loss of HD-tuin function resulted in the accumulation of hyperacetylated histones (Bourque et al., 2011; Ding et al., 2012), it is more likely that HD-tuins interact with RPD3-like HDAs and recruit them to the DNA (Luo et al., 2012a; Luo et al., 2012b). Treatment of seeds with the RPD3-like HDAs inhibitor trichostatin A (TSA) results in 90% inhibition of seed germination, concluding that histone deacetylation is required for processing seed germination (Tanaka et al., 2008). KO-mutant analysis revealed, that HD2A and HD2C play opposing functions in seed germination. While HD2A restrains germination, HD2C enhances germination (Colville et al., 2011). A combination of associated mapping and transcriptomics led to the identification of HD2B as a genetic factor associated with seed dormancy (Yano et al., 2013). However, little is known about the underlying precise mechanism of the HDAs in seed germination.

In this study, we provided hints that HD2A and HD2B were recruited by HSI2 and HSL1 and function redundantly in regulating seed dormancy by affecting the *DOG1* expression. Silencing of HD2A and HD2B leads to hyperacetylation of the *DOG1* locus, consequently, causing a strong accumulation of *DOG1* transcripts. *hd2ahd2b* seeds displayed abnormal phenotypes, but wild-type phenotypes could be partly restored by additional knock-out of *DOG1*. Transcriptome analysis revealed that the transcription of many seed storage-related genes is significantly changed in *hd2ahd2b*. Taken together, these data suggest that transcription repressors HSI2 and HSL1 might recruit HD2A and HD2B to repress *DOG1* expression during seed maturation and germination, contributing to seed normal development, and promoting seed germination in Arabidopsis.

## Materials and methods

### Plant materials and growth condition

The *Arabidopsis thaliana* ecotype Columbia-0 (Col-0) or mutants in the Col-0 background were used in all experiments. The T-DNA insertion lines GABI_355H03 (*hd2a*), Sail_1247_A02 (*hd2b*), Salk_039784 (*hd2c*), GK_379G06 (*hd2d*), and SM_3_20886 (*dog1-4*) were described previously (Luo et al., 2012a; Fedak et al., 2016; Li et al., 2017) and were verified by PCR on genomic DNA using gene-specific primers (**Supplemental Table S1**). The double mutant *hd2ahd2b*, *hd2ahd2c*, *hd2ahd2d*, *hd2bhd2c*, *hd2bhd2d*, *hd2chd2d*, and the triple mutants *hd2ahd2bdog1-4* were produced by crossing. Homozygous lines were isolated by genotyping with gene-specific primers (**Supplemental Table S1**). The HD2A and HD2B complementation lines were gifts from Ton Bisseling (Li et al., 2017). Seeds were sown in moist soil mixed with sand in a ratio of 10:1 and cultivated in the growth chambers under long-day conditions (14 h light/10 h dark and 20°C/18°C, respectively) or short-day conditions (10 h light/14 h dark and 20°C/16°C, respectively). The Arabidopsis plants used for seed production were grown first under short-day conditions for 4 weeks before being transferred to long-day conditions for flowering. The seeds were harvested and stored in the dark at room temperature.

### HDA activity measurement of total protein extracts

Measurements of HDA activity of *Arabidopsis* tissue protein extracts were performed by a fluorescence-based method adapted from Wegener et al. (Wegener et al., 2003a; Wegener et al., 2003b) and (Nott et al., 2008). 150 mg of ground deep-frozen plant material per replicate was transferred to a pre-cooled Lysing Matrix D tube (MP Biomedicals, Santa Ana, California, USA) and homogenized for 1 min at full speed in a FastPrep^®^-24 homogeniser (MP Biomedicals). After placing the tubes on ice, 300 µl homogenization buffer (50 mM Tris-HCl pH 7.0, 1 M D-Glucose, and 1x protease inhibitor cocktail) was added and the samples were again homogenized for 30 sec. The supernatant was transferred to 1.5 ml microcentrifuge tubes and centrifuged for 10 min at 25,000*g* and 4°C to remove cell debris. The protein concentration of the supernatant was determined according to Bradford (Bradford, 1976) and adjusted to a concentration of 1.2 µg/µl with homogenization buffer. HDA activity of the supernatant was assayed in 30 µl fractions per replicate in a flat-bottom 96-well black microtiter plate. 100 µM BOC-(acetyl) Lys-AMC (Bachem, Bubendorf, Switzerland) in 25 µl HDA reaction buffer (25 mM Tris-HCl, pH 8.0, 137 mM NaCl, 2.7 mM KCl and 1 mM MgCl_2_) was added. After incubation for 2 h at 37°C, 10 mg/ml trypsin and 1 µ M TSA in 60 µl, HDA stopping buffer (50 mM Tris-HCl, pH 8.0, 100 mM NaCl) was added per well and fluorescence output, representing HDA activity, was measured after 20 min incubation at 30°C at λ 380nm_Excitation_ and 440nm_Emission_.

### Co-IP analysis

For Co-IP analysis, the *35Spro : HSI2-myc* and *35Spro : HSL1-myc* constructs were transiently expressed in *35Spro : HD2A-GFP* and *35Spro : HD2B-GFP* protoplast. After brief centrifugation (100*g*, RT, 3 min), the supernatant was removed and total protein was extracted by re-suspending and disrupting the protoplast with 1 ml of extraction buffer (50 mM Tris-HCl, pH 8, 1 mM PMSF, 5% glycerol, 150 mM NaCl, 0.1% Nonidet P-40, 5 mM MgCl_2_, 1 mM DTT, and 1x protease inhibitor cocktail). After gentle shaking for 1 h at 4°C, the sample was centrifugated at 12,000*g* for 10 min. To purify GFP-tagged proteins, the supernatant was incubated with 25 μl GFP-Trap agarose beads (Nano tag; catalog no. N0510) at 4°C overnight by gentle rotation. After washing with extraction buffer four times, proteins were eluted with 50 μl 2x SDS sample buffer and analyzed by immunoblotting using an anti-Myc antibody.

### ChIP-qPCR assays

The ChIP-qPCR assay was performed as previously described (Bowler et al., 2004). The chromatin was extracted from 24 h imbibed WT and *hd2ahd2b* seeds and from 10 d old 35Spro: HD2B-GFP seedlings. The seeds were imbibed at room temperature under the dark, and at that time point, no germination was visible. About 1 g of imbibed seeds and 2 g of seedlings were cross-linked in cross-linking buffer (400 mM sucrose, 10 mM Tris-HCl pH 8.0, 5 mM β-mercaptoethanol, 1% formaldehyde) by vacuum infiltration for 1 h and 10 min, respectively. Cross-linking was stopped by adding glycine to an end concentration of 0.125 M and additional vacuum infiltration for 5 min. The cross-linked plant materials were washed two times with ice water, dried with paper towels, and ground in liquid nitrogen to a fine powder. The chromatin was extracted with 20 ml extraction buffer (400 mM sucrose, 10 mM Tris-HCl pH 8.0, 5 mM β-mercaptoethanol, 1x protease inhibitor cocktail) by gentle shaking for 20 min and pelleted by centrifugation at 4000g for 25 min at 4°C. The pellet was washed with nuclei washing buffer (20 mM Tris/HCl, pH 7.4, 25% glycerol, 2.5 mM MgCl_2_, 0.2% Triton x-100), re-suspended in 600 μl nuclei sonication buffer (50 mM Tris-HCl pH 8.0, 10 mM EDTA pH 8.0, 1% SDS, 1x protease inhibitor cocktail) and the chromatin was sheared to 200–1,000 bp by sonication. the nuclei were transferred into 1.5 ml Bioruptor Microtubes (Cat No. C30010016), and 15 cycles with 30 sec ON/OFF was used with Bioruptor^®^ Pico ultrasonic bath and Covaris E220 Evolution. After centrifugation for 10 min at 12,000g and 4°C the supernatant was directly used for immunoprecipitation with specific antibodies. For H3K9ac, H4K5ac, and H4ac analysis, the antibodies (anti-H4ac, anti-H4K5ac, anti-H3K9ac) were coupled to the magnetic protein G beads by incubating at 4°C on a rotation platform overnight. Afterward, 100 μl sonicated chromatin was mixed with antibody-magnetic protein G beads and incubated overnight at 4°C on a rotating platform. The beads were sequential washed with low salt buffer (0.1% SDS, 1% Triton X-100, 2 mM EDTA, 20 mM Tris-HCl pH 8.0, 150 mM NaCl), high salt buffer (0.1% SDS, 1% Triton X-100, 2 mM EDTA, 20 mM Tris-HCl pH 8.0, 500 mM NaCl), LiCl buffer (0.25 M LiCl, 1% NP-40, 1% sodium deoxychlorate, 1 mM EDTA, 10 mM Tris-HCl pH 8.0), and TE buffer (10 mM Tris-HCl pH 8.0, 1 mM EDTA). And the beads were washed twice with each buffer. Finally, the chromatin was eluted with 400 μl of elution buffer (1% SDS, 100 mM NaHCO_3_) by incubation for 20 min at 65°C, and the chromatin de-crosslinking was performed at 65°C for over 6 h after adding 16 μl of 5 M NaCl. After being treated with proteinase K and RNase, DNA was purified by phenol-chloroform method, eluted with dH_2_O, and quantified for qPCR. For anti-GFP analysis, the GFP-Trap agarose beads were used instead of antibody-coupled magnetic protein G beads, and analysis was performed with the same procedure as above.

### Bimolecular fluorescence complementation assay

For bimolecular fluorescence complementation assays, the ORF of *HD2A*, *HD2B*, *HSI2*, and *HSL1* (without stop codon) were transferred into the pDONOR221 vector by Gateway Cloning and subsequently shifted into the pBiFCt-2in1-NN vector (LR reactions) according to the described (Grefen and Blatt, 2012). Then, the constructs were transferred into Arabidopsis protoplasts by PEG transformation as described above. After incubation for 16 h to 24 h in the dark, the YFP fluorescence signal was monitored using a laser scanning confocal microscope (Leica TCS SP8 confocal).

### Determination of ABA and GA3

The endogenous ABA and GA3 contents were measured with Agilent 1290 Infinity II-6470 triple quadrupole LC/MS/MS System according to the previously reported method (Liu et al., 2021) with minor modifications. Briefly, 0.1 g dry seeds and 0.3 g 24 h imbibed seeds were ground into a fine powder with liquid nitrogen and were transferred into a 2 ml microtube containing 1 ml ethyl acetate. The samples were vortexed and incubated for 30 min at 4°C on a shaker. Afterward, the samples were centrifuged at 12,000*g* for 10 min at 4°C and the supernatant was transferred into a 1.5 ml tube and dried (speed vac). The residue was re-dissolved in 200 μl of 50% methanol and filtered through a 0.22 μm filter for sample loading. For each sample, 100 μl methanol solution was subjected to LC-MS/MS analysis. ABA (yuan ye biotech, catalog no. B50724) and GA3 (yuan ye biotech, catalog no. B20187) were used as authentic reference standards. All determinations were performed in triplicate.

## Result

### Silencing of HD2A and HD2B caused deeper seed dormancy

**Figure 1:**
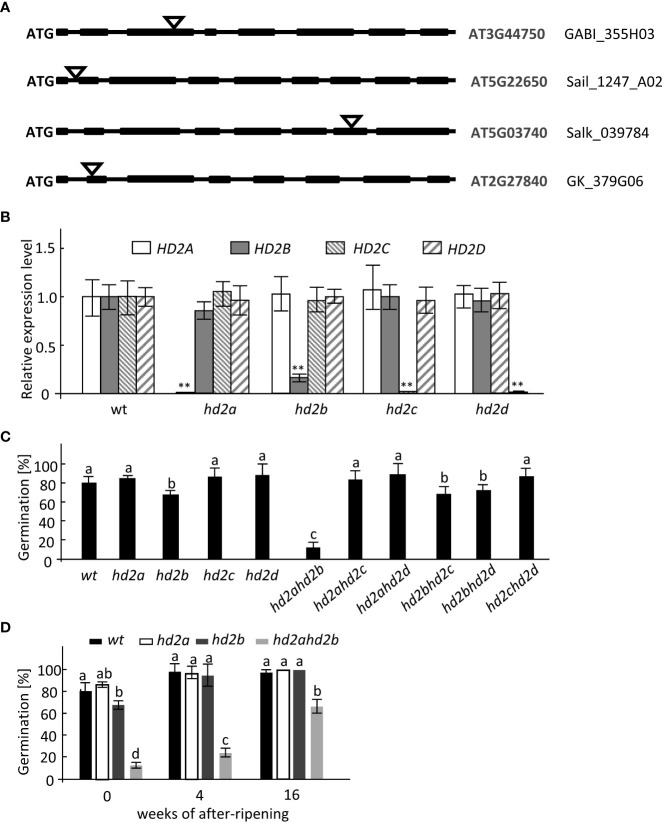
*hd2ahd2b* double KO line shows enhanced seed dormancy. **(A)** Gene structure of HD2A, HD2B, HD2C, and HD2D and T-DNA insertion sites are shown. Exons, introns, and T-DNA insertions are represented by black boxes, lines, and triangles, respectively. **(B)** RT-qPCR analysis of *HD2A*, *HD2B*, *HD2C, and HD2D* expression levels in WT and *HD2s* single mutant lines of 24h-imbibed seeds. RT‐qPCR signals were normalized to *UBQ5* expression levels. **(C)** Germination percentage of freshly harvested wild-type and HD2s mutant seeds. The seeds were sown on water-saturated filter paper. After 3 days of incubation, the germination rates were analyzed. **(D)** Germination percentage of non-stratified wild-type, *hd2a*, *hd2b*, and *hd2ahd2b* seeds after different periods of dry storage. The seeds were sown on water-saturated filter paper. After 3 days of incubation, the germination percentages were analyzed. Data represented are averages ± SE of three independent experiments. Asterisks in **(B)** indicate a significant difference between the mutant and wild type (**P < 0.01). Lowercase letters indicate significant differences compared with the wild type in **(C)** (P < 0.01) and significant differences (P < 0.01) between different samples in **(D)**, One-Way ANOVA (Tukey-Kramer test) analysis was performed.

To investigate the precise functions of HD2s in Arabidopsis germination, we analyzed seed germination of all four HD2s T-DNA insertion lines of Col-0 background under long-day conditions, designated as *hd2a*, *hd2b*, *hd2c*, and *hd2d* (**Figure 1A**). Reduced transcript accumulation in 24h-imbibed seeds was confirmed by qRT-PCR. The results showed that nearly no transcripts of *HD2A*, *HD2C*, and *HD2D* were detectable (**Figure 1B**), whereas transcription of HD2B is reduced by approx. 80% (**Figure 1B**). Moreover, double mutants were generated by crossing the HD2 T-DNA insertion lines to determine, if functional redundancy is existing in seed germination among the different HD2s. Freshly harvested seeds were used for seed dormancy analysis. After three days of incubation, between 80% and 85% of WT, *hd2a*, *hd2c*, and *hd2d* seeds were germinated, whereas, only 70% of *hd2b* seeds germinated (**Figure 1C**). Among the six double mutant lines, *hd2ahd2b* double mutant showed a strongly enhanced seed dormancy phenotype, only around 15% germinated (**Figure 1C**, **Supplemental Figure S1**). These results suggest that HD2A is at least partly functional redundancy to HD2B in activating seed germination. After 4 weeks of dry storage at 4°C, seeds of WT and *hd2a*, and *hd2b* single mutant lines germinated almost 100%, whereas *hd2ahd2b* seeds germinated only to 25% and even after extended storage of 16 weeks only to 65% (**Figure 1D**). These results indicated that HD2A and HD2B play an important role in promoting seed germination.

### *HD2A* and *HD2B* expression patterns during seed maturation and imbibition

**Figure 2:**
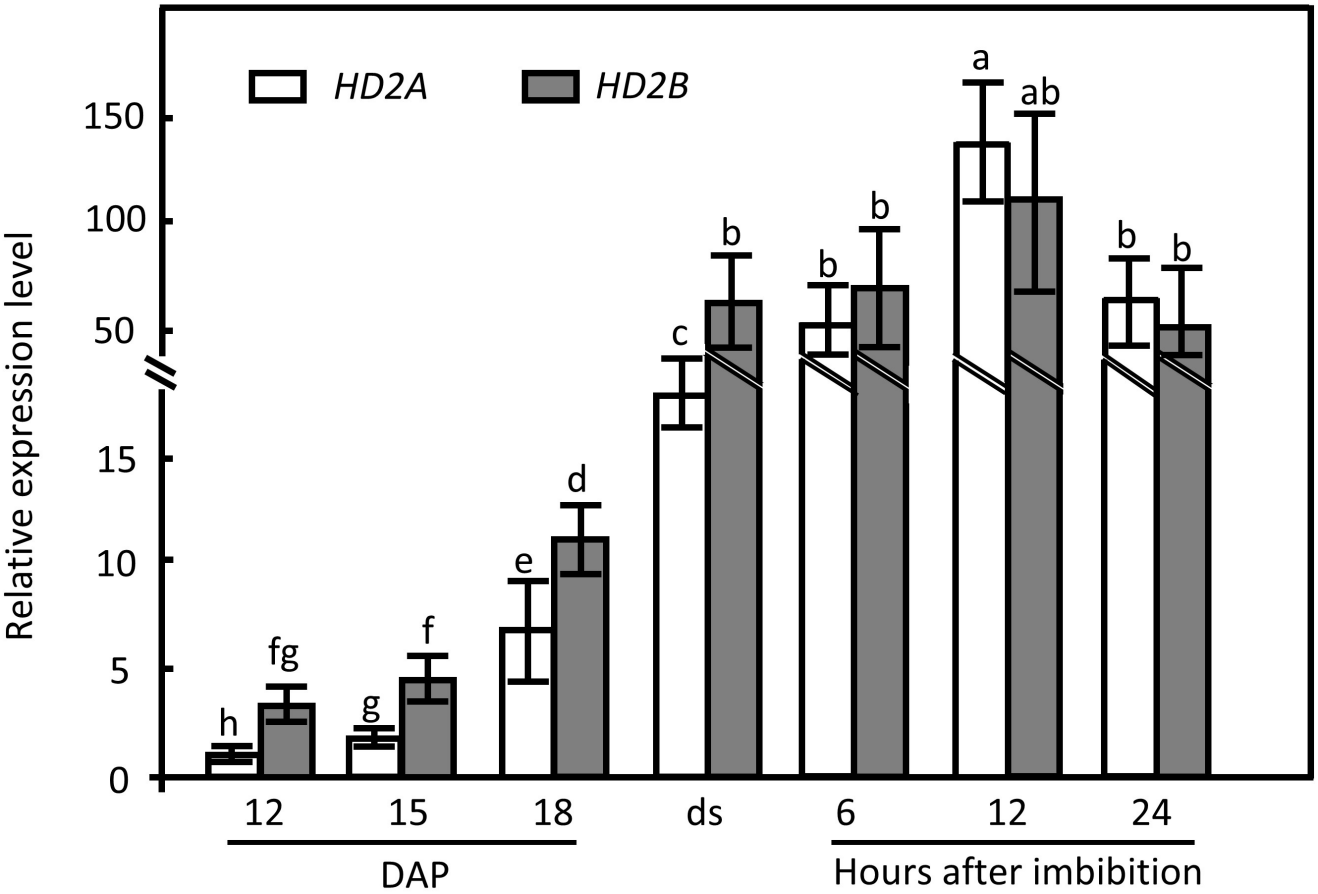
*HD2A* and *HD2B* expression pattern during maturation and imbibition of wild-type seeds. The expression of *HD2A* and *HD2B* at different seed developmental and imbibition stages were analyzed by RT-qPCR. Expression of *UBQ5* was used for normalization. The expression was analyzed 12 days after pollination (DAP), 15 DAP, 18 DAP, and in freshly harvested dry seeds (ds). Moreover, mature seeds were analyzed at 6 h, 12 h, and 24 h of imbibition at 20°C under the light. Three biological replicates were performed. The average ( ± SD) values are shown. Lowercase letters indicate significant differences (P < 0.05) between the different values. One-Way ANOVA (Tukey-Kramer test) analysis was performed.

To unveil the underlying function of HD2A and HD2B in seed dormancy release, their temporal expression pattern during seed maturation and imbibition was examined by RT-qPCR. *HD2A* has an analogous expression pattern as *HD2B*, rapidly increasing from 12 d after pollination (DAP) and reaching the highest expression level in 12 h imbibed seeds (**Figure 2**). In stored seeds, the expression level of *HD2B* is significantly higher than that of *HD2A*. In general, imbibed seeds displayed significantly higher expression levels of both genes than maturating seeds (**Figure 2**). These results imply a function of HD2A and HD2B in seed dormancy establishment as well as dormancy release.

### HD2A and HD2B regulate seed dormancy *via* an ABA signal transduction pathway

**Figure 3:**
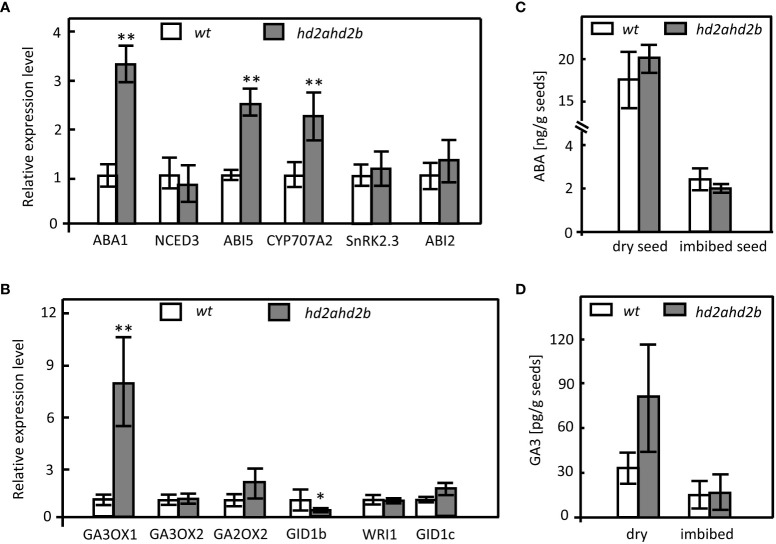
Expression of genes involved in ABA and GA metabolism and signal transduction pathways and endogenous ABA and GA3 levels in WT and hd2ahd2b. Changes in transcript levels of genes involved in ABA **(A)** and GA **(B)** biosynthesis, catabolism, and signal transduction were analyzed in 24 himbibed seeds analyzed by RT-qPCR. Expression of *UBQ5* was used for normalization. ABA **(C)** and GA3 **(D)** content in dry seeds and seeds imbibed for 24h. The phytohormone content of the seeds was determined by LC-MS. Error bar represents the ± SD of 3 biological replicates. Asterisks in **(A, B)** indicate a significant difference between *hd2ahd2b* and WT based on One-Way ANOVA (Tukey-Kramer test) (*P < 0.05, **P < 0.01).

To analyze, if HD2A and HD2B regulate seed germination *via* ABA and/or GA biosynthesis and signal transduction pathways, transcripts of genes involved in ABA and GA3 biosynthesis/catabolism/signaling have been quantified (**Figures 3A, B**). The transcription of *ABA1* and *CYP707A2*, involved in ABA biosynthesis and catabolism, respectively, was increased in *hd2ahd2b* (**Figure 3A**), whereas the expression of *NCED3*, *SnRK2.3*, and *ABI2*, which are related to ABA synthesis and ABA signal transduction, was not significantly different between *hd2ahd2b* and WT (**Figure 3A**). In contrast, expression of *ABI5*, another ABA signal transduction-related gene, was enhanced in *hd2ahd2b* (**Figure 3A**).

Regarding genes involved in GA biosynthesis, the expression of *GA3OX1* but not that of *GA3OX2* was upregulated in the *hd2ahd2b* seeds in comparison to WT. Moreover, the expression of *GA2OX2*, a gene required for GA catabolism, was not changed (**Figure 3B**). Furthermore, the analysis of the GA-repressed gene *WRI1* and the gibberellin receptors encoding genes *GID1b* and *GID1c* revealed that *GID1b* was downregulated in *hd2ahd2b*, whereas the expression of *WRI1* and *GID1c* was not significantly different in WT and *hd2ahd2b* seeds (**Figure 3B**).

Since the expression of at least a few genes related to ABA and GA3 biosynthesis/catabolism/signaling is affected in *hd2ahd2b*, we determined the content of ABA and GA3 in WT and *hd2ahd2b* seeds. ABA and GA3 content is lower in 24 h imbibed seeds in comparison to dry seeds of WT and *hd2ahd2b* plants (**Figures 3C, D**). Surprisingly, the amount of ABA and GA3 was not significantly different neither in dry nor in imbibed seeds of *hd2ahd2b* and WT (**Figures 3C, D**).

In conclusion, although ABA and GA3 content is not significantly affected in *hd2ahd2b* dry and imbibed seeds, the upregulation of ABI5 indicated that HD2A and HD2B somehow could function in the ABA signaling pathway to induce seed dormancy.

**Figure 4:**
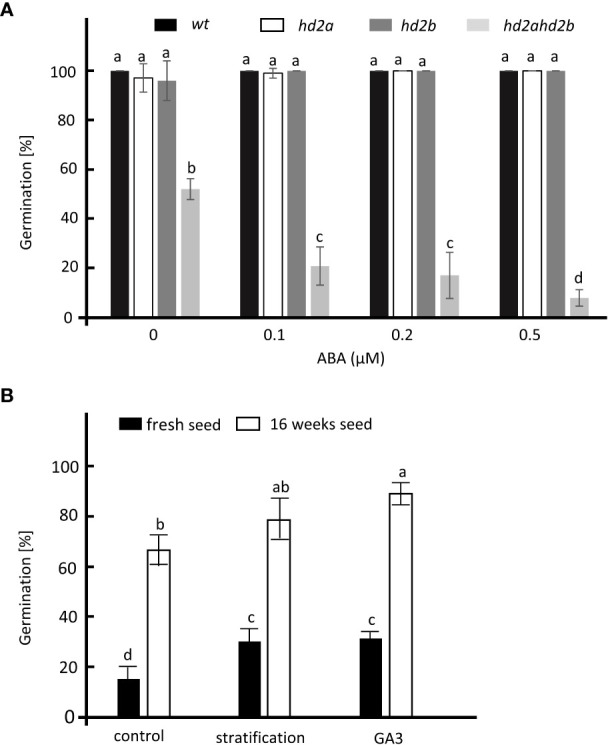
Germination analysis of different *hd2* lines. **(A)** Germination of WT, *hd2a*, *hd2b*, and *hd2ahd2b* seeds in presence of different concentrations of ABA. Sixteen weeks after-ripened seeds were imbibed on ½ MS plate in the presence of 0, 0.1, 0.2, and 0.5 µM of ABA. The germination percentage was scored after 3 days. **(B)** Germination of fresh and 16 weeks old *hd2ahd2b* seeds after stratification and GA3 treatments. For stratification, seeds were placed on water-saturated filter paper and stratified for 3 days at 4°C before transferring to the growth chamber. For GA3 treatment, non-stratified seeds were sown on filter paper saturated with 100 μM of GA3. The germination percentages were scored 3 days after incubation. Statistics: The error bar represents the ± SD of at least 3 biological replicates. Lowercase letters indicate significant differences (P < 0.05) between the different values. One-Way ANOVA (Tukey-Kramer test) analysis was performed.

Since mutants with a seed dormancy phenotype are usually hypersensitive to ABA (Zhao et al., 2015; Née et al., 2017), we analyzed the germination of fully after-ripened *hd2ahd2b* and WT seeds in presence of different concentrations of ABA. *hd2ahd2b* displayed significantly reduced seed germination with increasing concentrations of ABA, whereas no effect was observed in seed germination of WT and the single mutant lines, demonstrating that only the double mutant *hd2ahd2b* is hypersensitive to ABA (**Figure 4A**). A time course experiment demonstrated that *hd2ahd2b* seeds are 100% viable, but showed delayed germination already in absence of ABA (**Supplemental Figure S2**). Moreover, we tested germination of *hd2ahd2b* in presence of 100 µM of GA3 and after stratification at 4°C for 3 days. Both treatments slightly promoted the germination of freshly harvested and completely ripened *hd2ahd2b* seeds (**Figure 4B**).

### HD2A and HD2B promote seed germination *via* repressing *DOG1*

**Figure 5:**
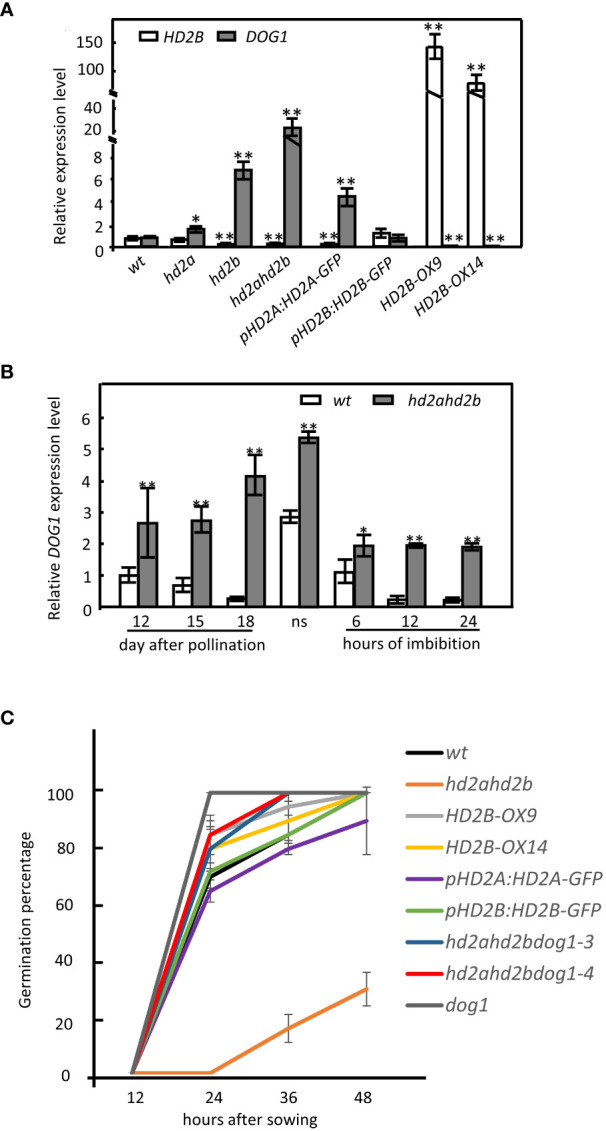
*HD2B* negatively regulates *DOG1* expression and promotes seed germination. **(A)** RT-qPCR analyses of *HD2B* and *DOG1* expression in 24h imbibed seeds of WT, *hd2a*, *hd2b*, *hd2ahd2b, HD2B-OX and* complementation lines *pHD2A: HD2A-GFP* and *pHD2B: HD2B-GFP*. Seeds were stored at room temperature for 16 weeks before imbibition. **(B)** *DOG1* expression pattern during seed maturation and imbibition in WT and *hd2ahd2b*. “ns” indicate freshly harvested seeds. Expression of *UBQ5* was used for normalization. The error bar represents the ± SD of 3 biological replicates. Asterisks in **(A)** indicate a significant difference between the different lines compared with WT. Asterisks in **(B)** indicate significant differences between the different samples. One-Way ANOVA (Tukey-Kramer test) analysis was performed, (*P < 0.05, **P < 0.01). **(C)** Germination percentage of the lines with different *DOG1* expression levels. Fully after-ripened seeds were stratified for 3 days at 4°C before incubation in the growth chamber. The error bar represents the ± SD of 3 biological replicates.

Besides ABA, the protein DELAY OF GERMINATION 1 (DOG1) is an essential regulator of seed dormancy (Bentsink et al., 2006; Nakabayashi et al., 2012; Huo and Bradford, 2016). The gradually elevated expression of *HD2A* and *HD2B* during seed maturation and the fact that the *hd2ahd2b* double mutant line displays a “hypersensitive to ABA” germination phenotype, let us assume that HD2A and HD2B affect the expression of *DOG1*. Therefore, we analyze the relative expression level of *DOG1* in imbibed seeds of WT, the single mutant lines *hd2a* and *hd2b*, the double mutant line *hd2ahd2b*, the double mutant line either complemented with *HD2A-GFP* (*pHD2A: HD2A-GFP)* or *HD2B-GFP* (*pHD2B: HD2B-GFP)* and the two *HD2B* overexpression lines *HD2B-OE9*, *HD2B-OE14*. *DOG1* transcription level in the *pHD2B: HD2B-GFP* line was comparable to that of WT seeds (**Figure 5A**). In contrast, the *DOG1* expression level was significantly decreased in the *HD2B* overexpression lines and elevated in the line with a lower *HD2B* transcription level (**Figure 5A**). Notably, in seeds of the *hd2ahd2b* double mutant, the *DOG1* expression level was increased more than 20 times in comparison to WT, while in *hd2a* and *hd2b* expression of *DOG1* was only two- and six-fold increased, respectively (**Figure 5A**). Surprisingly, the expression level of *DOG1* in the *pHD2A: HD2A-GFP* complementation line is significantly higher than in the WT (**Figure 5A**). In conclusion, higher *HD2B* expression correlates with lower *DOG1* expression indicating that HD2B may repress *DOG1* expression during seed germination. Moreover, both, HD2A and HD2B functions are essential for regulating the expression of *DOG1* during seed germination.

We further analyzed the dynamics of *DOG1* expression in *hd2ahd2b* during seed maturation and imbibition. We observed that the *DOG1* mRNA accumulation decreased from 12 DAP until seed maturation, which is consistent with the reported data in the Col-0 background (Zhao et al., 2015). Then, *DOG1* expression increased rapidly in dry seeds and quickly vanished after seed imbibition (**Figure 5B**). Different from the expression pattern in WT, the *DOG1* transcript level in the *hd2ahd2b* double mutant increased during seed maturation, peaked in dry seed, and decreased during imbibition. Interestingly, after an initial significant decrease at beginning of imbibition, *DOG1* expression in *hd2ahd2b* seeds did not vanish as observed in WT seeds but remained at a relatively stable level over at least 24 h (**Figure 5B**). In general, the *DOG1* expression level in *hd2ahd2b* is significantly higher during seed development and imbibition in comparison to WT (**Figure 5B**), concluding that freshly harvested seeds of the *hd2ahd2b* line might accumulate more DOG1 than WT seeds. To further get evidence for a coordinated function of HD2A/HD2B and DOG1 in seed germination, we analyzed seed germination of Arabidopsis lines with different *HD2A/HD2B* and *DOG1* expression levels. Compared to all other lines analyzed, the seeds of the *hd2ahd2b* double mutant with the highest *DOG1* mRNA level showed significantly lower (delayed) germination. Interestingly, this reduced germination phenotype is restored in the complementation line *pHD2B: HD2B-GFP* and partially in *pHD2A: HD2A-GFP*. Although the *dog1* mutant and the *HD2B* overexpression lines *HD2B-OX9* and *HD2B-OX14* showed reduced expression of *DOG1* and a similar percentage of germination as WT seeds after 2 days of incubation, the percentage of germination of WT is slightly delayed in comparison to that of *dog1* and both *HD2B* overexpression lines (**Figure 5C**).

To demonstrate the functional relationship between HD2A, HD2B, and DOG1, we crossed *hd2ahd2b* and *dog1-3* and *dog1-4* to generate *hd2ahd2bdog1-3* and *hd2ahd2bdog1-4* homozygous plants. Almost all triple mutants’ seeds germinated after incubation for 48 h, indicating a none dormancy phenotype similar to *dog1* mutants (**Figure 5C**). In conclusion, the DOG1 function is responsible for *hd2ahd2b* mediated seed dormancy. All these results suggested that HD2A and HD2B may promote seed germination by inhibiting *DOG1* expression.

### HD2A and HD2B deacetylate *DOG1*

**Figure 6:**
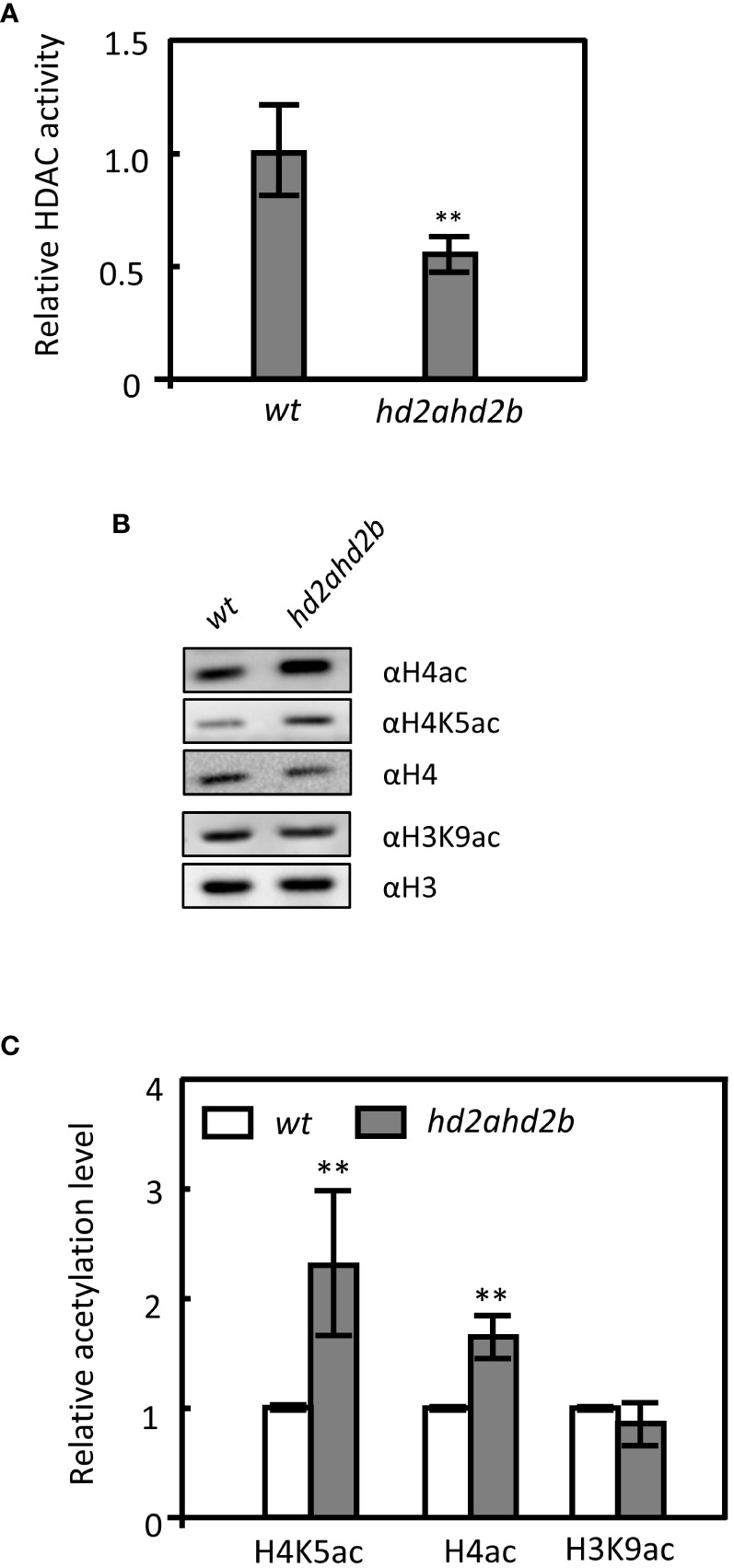
Loss of HD2A and HD2B function caused global hyperacetylation on H4 and H4K5 in Arabidopsis. **(A)** Relative HDA activity in protein extracts of WT and *hd2ahd2b* plants. The total soluble protein of 10 days old seedlings was extracted and the HDA activity was measured using a modified fluorometric assay. **(B)** Detection of histone acetylation levels in WT and *hd2ahd2b* mutant seeds by immunoblotting. Four independent replicates were performed with similar results. **(C)** The intensities of the signals of the immunoblot were quantified using the ImageJ software. Four biological replicates (mean ± SD) were normalized to the signals of H4 (H4ac and H4K5ac) and H3 (H3K9ac). The WT signals were set to 1 for each analyzed histone modification. Error bars represent the ± SD of 4 biological replicates. Asterisks indicate a significant difference between WT and *hd2ahd2b.* One-Way ANOVA (Tukey-Kramer test) analysis was performed, (**P < 0.01).

HD2A and HD2B are annotated as HDAs, however, their exact biochemical functions in Arabidopsis are still unknown. To confirm that HD2A and HD2B are required for histone deacetylation, the relative HDA activity in 10 days old seedlings of *hd2ahd2b* and WT plant was measured. *hd2ahd2b* displayed a 45% reduction in total HDA activity compared to WT (**Figure 6A**), suggesting that HD2A and HD2B are required for HDA activity in Arabidopsis.

**Figure 7:**
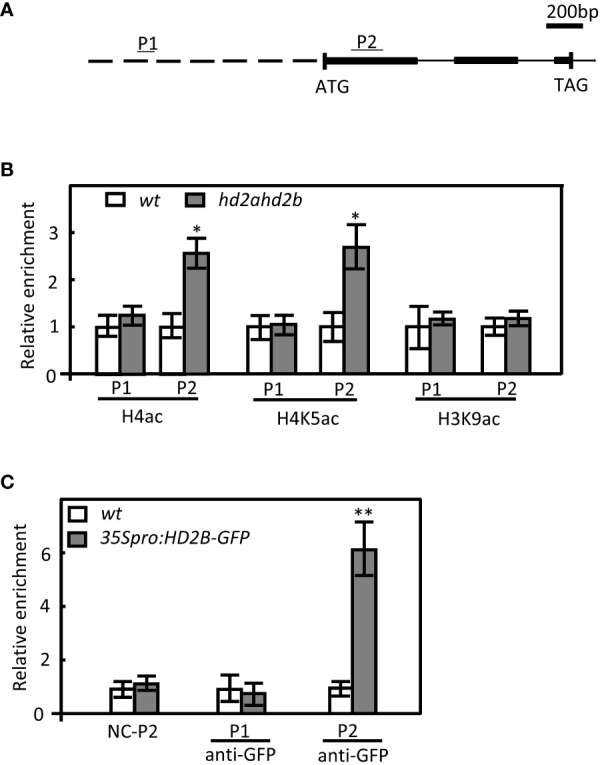
Enhanced H4 and H4K5 acetylation levels in *hd2ahd2b* seeds at *DOG1*. **(A)** Schematic illustration of the *DOG1* genomic region examined by ChIP-qPCR. Promoter, exons, and introns are represented by the dashed line, black boxes, and continuous lines, respectively. The regions analyzed in the ChIP-qPCR are indicated above the gene structure as P1 and P2. **(B)** ChIP-qPCR analysis of H4, H4K5, and H3K9 acetylation of *DOG1*. Immunoprecipitated DNA was obtained from 24 h imbibed WT and *hd2ahd2b* seeds using the indicated specific antibodies against the analyzed histone marks. Specific primers for P1 and P2 were used. **(C)** ChIP-qPCR analysis of *35Spro: HD2B-GFP* complementation line with GFP antibody. Immunoprecipitated DNA was obtained from 10 days seedlings of WT and *35Spro: HD2B-GFP* with anti-GFP antibody. The relative amount of PCR products using P1 and P2 specific primers were quantified and normalized to internal control (*s16*). The values shown are means ± SD. Error bars represent the SD of 3 biological replicates for each ChIP-qPCR experiment. Asterisks in indicate a significant difference (*P < 0.05, **P < 0.01).

Furthermore, the global acetylation levels of H4, H4K5, and H3K9 in germinated seeds of WT and *hd2ahd2b* double mutant were analyzed. In *hd2ahd2b* a 1.4-fold and 2.2-fold enhanced H4ac and H4K5ac level, respectively, was detected, but no significant difference was observed in the H3K9ac level (**Figures 6B, C**). Subsequently, we analyzed whether the acetylation level at the *DOG1* promoter and the *DOG1* coding region is altered in the *hd2ahd2b* line in comparison to WT. We performed chromatin immunoprecipitation quantitative PCR (ChIP-qPCR) on 24 h imbibed seeds of WT and *hd2ahd2b* using specific anti-H3K9ac, anti-H4ac, and anti-H4K5ac antibodies. A 103 bp promoter region P1, 1064 bp upstream of the transcription start site (TSS), and a 189 bp coding region P2, 157 bp downstream of TSS, were amplified with specific primers (**Figure 7A**). Loss of HD2A and HD2B function enhanced the acetylation level of H4ac and H4K5ac at the coding region P2 of *DOG1*, but not at the promoter region P1 (**Figure 7B**). In contrast, the H3K9ac level did not significantly change in *hd2ahd2b* in comparison to WT, neither in the *DOG1* promoter region P1 nor the *DOG1* coding region P2 (**Figure 7B**). To check, whether HD2B directly binds to the *DOG1* locus, we performed ChIP-qPCR on 24 h imbibed seeds of WT and *hd2ahd2b* complemented with *35Spro: HD2B-GFP* using an anti-GFP antibody. Arabidopsis intergenic region between AT5G43175 and AT5G43180 was selected as the negative control. Fragments corresponding to the *DOG1* coding region P2 were significantly enriched concluding that HD2B-GFP binds to *DOG1*. No enrichment was detected in the promoter region P1 and the negative control. In sum, these data indicate that the up-regulation of *DOG1* in *hd2ahd2b* is at least partly due to increased histone acetylation at the *DOG1* coding region P2.

### HD2A and HD2B form hetero-oligomers and may be recruited by HSL1and HSI2 to regulate *DOG1* expression through deposition of histone acetylation

HD2A and HD2B need to be recruited to the different DNA binding sites by different transcriptional regulators and form multi-protein complexes to modulate chromatin structure and consequently regulate gene expression in the different development stages (Liu et al., 2014). HSI2 and its homolog HSI2-like 1 (HSL1), also known as VAL1 and VAL2, respectively, repress *DOG1* expression by recruiting LIKE HETERCHROMATIN PROTEIN 1 (LHP1) and CURLY LEAF (CLF) for consequent deposition of H3K27me3 marks at *DOG1* locus (Chen et al., 2020).

**Figure 8:**
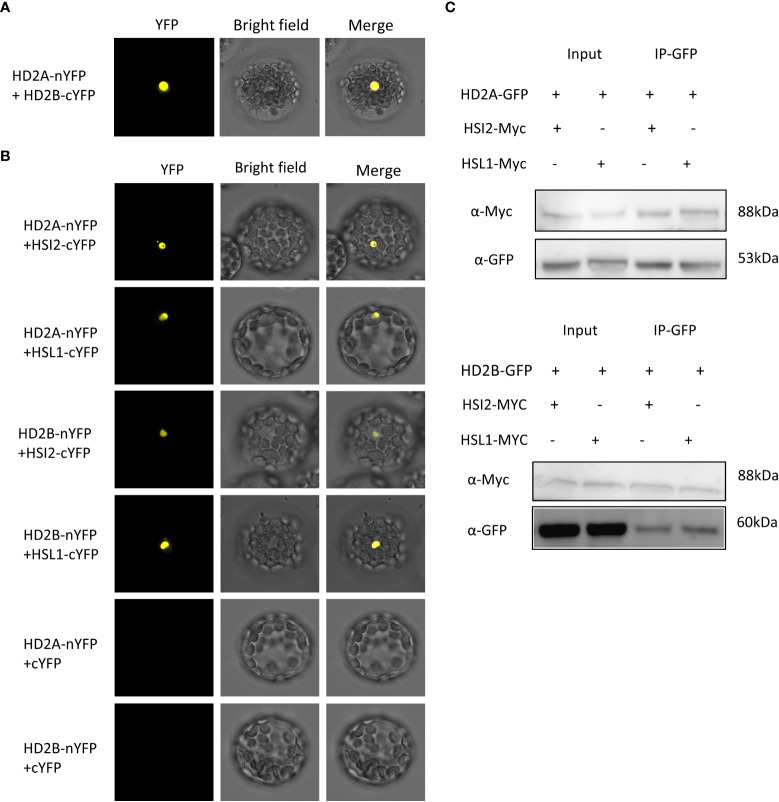
HD2A and HD2B interact with HSI2 and HSL1 *in vivo*. **(A)** Bimolecular fluorescence complementation (BiFC) showing protein–protein interactions between HD2A and HD2B. HD2A was fused to the N-terminus of YFP (nYFP) and HD2B was fused to the C-terminus of YFP (cYFP). Both constructs were co-transfected into Arabidopsis protoplast and visualized using a confocal microscope after cultivating for 24 hours at 25°C. **(B)** BiFC showing protein-protein interactions between HD2A, HD2B, HSL1, and HSI2 in Arabidopsis mesophyll protoplasts. HD2A and HD2B were fused to the N-terminus of YFP (nYFP) and HSL1 and HSI2 were fused to the C-terminus of YFP (cYFP). The constructs were co-transfected into Arabidopsis mesophyll protoplasts as indicated and visualized using a confocal microscope after cultivating for 24 hours at 25°C. As negative control, empty plasmids containing cYFP and HD2A or HD2B fused with nYFP were co-transfected into Arabidopsis mesophyll protoplasts. Bar, 20μm. **(C)** Co-immunoprecipitation assays demonstrating interactions between HD2A, HD2B, HSL1, and HSI2 *in vivo*. Myc-tagged HSL1 and HSI2 were transfected into the Arabidopsis mesophyll protoplast of HD2A-GFP and HD2B-GFP overexpression line. Total protein was extracted, HD2A-GFP and HD2B-GFP were immunoprecipitated with anti-GFP antibody and the immunoblot was detected with anti-GFP anti-Myc antibody.

We assumed that HD2A and HD2B are also recruited to *DOG1* by HSL1 and HSI2. The interaction between HD2A and HD2B was demonstrated by bimolecular fluorescence complementation (BIFC) (**Figure 8A**). To analyse the interaction of HD2A/HD2B with HSL1/HSI2, HD2A, and HD2B were fused to the N-terminus of YFP, and HSL1 and HSI2 were fused to the C-terminus of YFP. YFP signals were observed in the nucleus whenever HD2A or HD2B were co-expressed with HSL1 or HSI2 (**Figure 8B**). No signals were detected when HD2A and HD2B were co-transfected with the empty plasmid containing YC-YFP (negative control, **Figure 8B**). Co-IP confirmed the interactions observed in the BIFC assay, since both, HSI2 and HSL1, co-immunoprecipitated with HD2A-GFP and HD2B-GFP (**Figure 8C**). These results indicate that HD2A and HD2B could be recruited by HSI2 and HSL1 to the *DOG1* locus.

### HD2A and HD2B regulate seed development by affecting the expression of *DOG1*

Seed development comprises embryo morphogenesis and seed maturation (Baud et al., 2008). The phenotype of Arabidopsis cotyledons was determined during embryo morphogenesis and the maturation process ensures the embryo accumulates enough storage reserves, which are important for seed dormancy and desiccation tolerance establishment (Baud et al., 2008; Carrillo-Barral et al., 2020). The abnormal development of the seeds plays important role in seed dormancy (Focks and Benning, 1998; Debeaujon et al., 2018), and embryo dormancy and coat-imposed dormancy are the two major types of seed dormancy mechanisms (Bewley, 1997).

**Figure 9:**
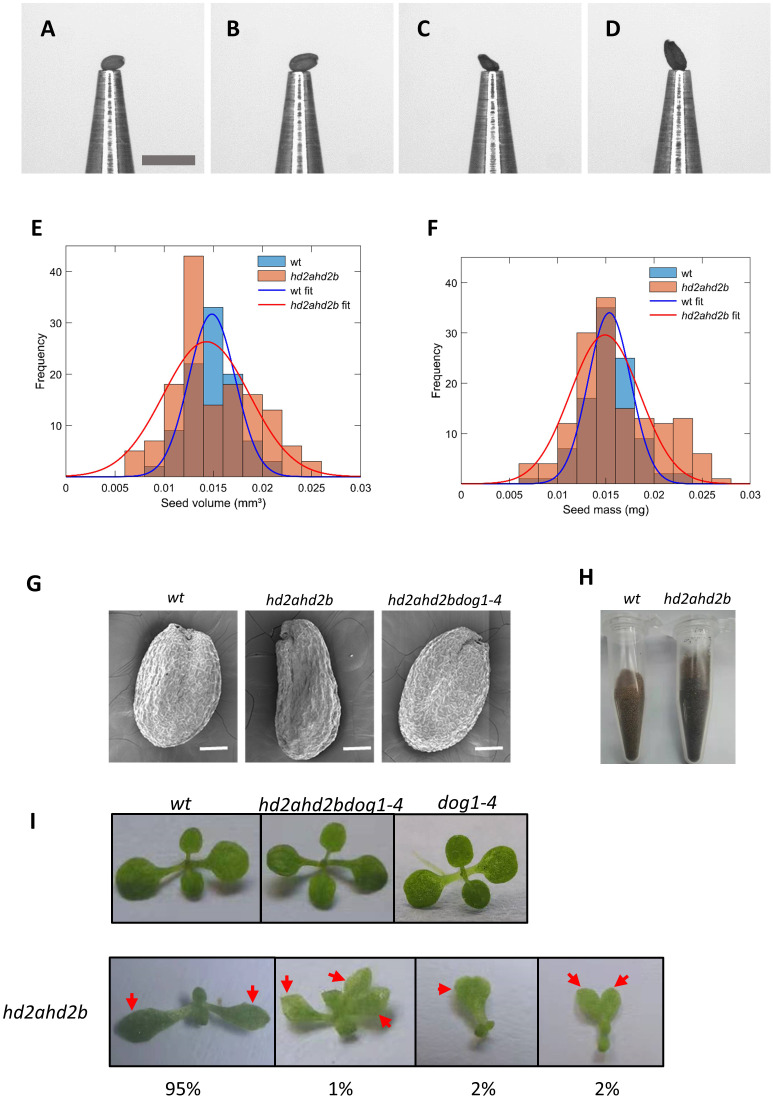
Seed and seedling phenotypes of WT and *hd2ahd2b* mutants. Example pictures of single seeds at the nozzle of the *pheno*Seeder. **(A, B)** small and large seed of Col-0 WT, respectively, **(C, D)** small and large seed of *hd2ahd2b*, respectively. Scale bar 1 mm. Seed traits of Arabidopsis genotypes Col-0 and *hd2ahd2b*. Frequency histograms of volume **(E)** and seed mass **(F)** with corresponding normal distribution fits. **(G)** Microscope images of WT, *hd2ahd2b*, and *hd2ahd2bdog1-4* mature dry seeds. **(H)** The color phenotype of WT and *hd2ahd2b* seeds. **(I)** The phenotype of WT, *hd2ahd2b*, and *hd2ahd2bdog1-4* seedlings 14d after germination. The numbers below the pictures indicate the observed frequency of each phenotype. Red arrows indicate the cotyledons.

Here we first found the displayed dysplastic cotyledons of *hd2ahd2b* seedling (tri-cotyledony, fused cotyledons, asymmetric cotyledons, and in the most extreme cases blurred border or junction between the petiole and the blade) (**Figure 9I**), which indicating abnormal embryo morphogenesis. Furthermore, the Arabidopsis WT and *hd2ahd2b* seeds were phenotyped using the *pheno*Seeder (Jahnke et al., 2016), which consists of a pick-and-place robot and several sensors, enabling measurement of seed traits such as mass, volume, density, length, width, and size (i.e., projected area) for individual seeds. Although *hd2ahd2b* seeds have an irregular surface, there were no relevant differences between mean traits of the genotypes (**Figures 9A–G**; **Table 1**), except that trait distributions of *hd2ahd2b* were wider and showed deviations from normal distributions for seed mass and volume (**Figures 9E, F**) (Bourque et al., 2011). We observed smooth testa, oval-shape, and brown color for WT seeds, whereas *hd2ahd2b* seeds displayed abnormal seed phenotype, with a wrinkled epidermis, irregular shape, and deeper testa color (**Figures 9G, H**). All those seed phenotypes are similar to the phenotype of mutants in seed maturation regulators and seed coat mutants, which could affect seed germination (Focks and Benning, 1998).

**Table 1:** Summary of mean measured seed traits of WT and *hd2ahd2b*.

		Mass	Volume	Density	Length	Width	Size
		(mg)	(mm³)	(mg/mm³)	(mm)	(mm)	(mm²)
**WT**	n	99	96	96	101	101	101
	Mean	0.0154	0.0149	1.044	0.499	0.326	0.126
	SD	0.0026	0.0024	0.034	0.036	0.024	0.014
***hd2ahd2b***	n	147	143	141	148	148	148
	Mean	0.0160	0.0152	1.062	0.524	0.319	0.128
	SD	0.0043	0.0041	0.062	0.055	0.032	0.019
	Cohen’s d	0.012	0.004	0.080	0.117	0.044	0.014

SD = standard deviation. Effect sizes of differences in means between genotypes were estimated by Cohen’s d value (d < 0.2 means no or very small effects).

It was reported that DOG1 was involved in seed development by affecting multiple aspects of seed maturation *via* genetic interaction with ABI3 (Dekkers et al., 2016). More important, The abnormal phenotypes of *hd2ahd2b* largely were recovered in *hd2ahd2bdog1* triple mutants (**Figure 9I**), suggesting a genetic interaction between *DOG1* and *HD2A/HD2B*. These findings demonstrate a possible underlying dormancy mechanism caused by the up-regulation of *DOG1* in *hd2ahd2b* lines, that HD2A and HD2B are involved in seed dormancy partly by regulating seed development.

### Transcriptome analysis of *hd2ahd2b* imbibed seeds and seedlings

**Figure 10:**
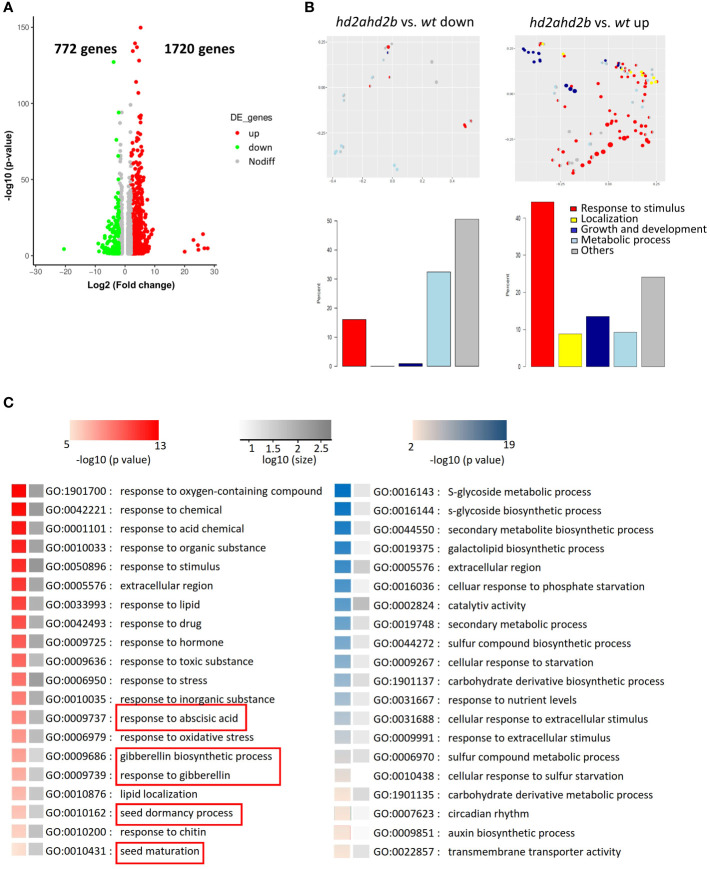
HD2A and HD2B function is required for the downregulation of genes involved in stress response and seed development in 10 days old seedlings. **(A)** RNA-seq analysis of 10 days old wt and *hd2ahd2b* seedlings. Volcano plots showing differentially expressed genes in *hd2ahd2b* seedlings in comparison to wt. Genes with an adjusted *P* value of < 0.05 and a log_2_ fold-change ≥2 or log_2_ fold-change ≤ -2 are highlighted in red and green, respectively. **(B)** Multi-dimensional scaling analysis of significantly enriched GO terms (adjusted p-value < 0.05) among the significantly up-regulated or down-regulated genes (adjusted p-value < 0.05) changed for *hd2ahd2b* vs. wt. Only GO terms from the biological process ontology are shown in the plot. Each circle corresponds to an enriched GO term. Its size is proportional to the number of differentially regulated genes assigned to the GO term. The enriched GO terms are arranged in two dimensions such that their distance approximately reflects how distinct the corresponding sets of differential genes are from each other, i.e. neighboring circles share a large fraction of genes. Each enriched GO term is colored by its membership in the top-level categories, which are grouped into five themes. If a GO term belongs to multiple top-level terms, a pie chart within the circle indicates the relative fraction of each theme. The total distribution of themes across all enriched GO terms is depicted in the bar plots below. **(C)** Significantly up- (red) and down-regulated (blue) enriched GO terms. The grey scale indicates the number of up-and-down-regulated genes in the corresponding enriched GO term. GO terms related to seed dormancy and germination are highlighted.

Our data suggest that HD2A and HD2B have important function in seed germination and seedling development. To get a general overview of the physiological processes, HD2A and HD2B are involved in, we performed an RNA-sequencing (RNA-seq) analysis of ten days old WT and *hd2ahd2b* seedlings and 24h imbibed seeds. Differentially expressed genes were defined based on a threshold of at least 2-fold change (P-value < 0.05). In seedlings, we found, that 1720 and 772 genes were up- and down-regulated in *hd2ahd2b*, respectively (**Figure 10A**; **Supplemental Table S2**), demonstrating the repressive function of plant-specific histone deacetylases (Wu et al., 2003; Zhou et al., 2004; Li et al., 2017; Chen et al., 2018). Interestingly, approx. 45% of the up-regulated genes (~775), but only 15% of the down-regulated genes (~120) have a function related to “response to stimuli” (**Figure 10B**). Moreover, significantly more genes related to “localization” and “growth and development” are up-regulated in *hd2ahd2b* seedlings (**Figure 10B**). The Gene Ontology enrichment analyses of 2492 differentially expressed genes revealed that within the up-regulated genes, genes related to “response to different chemicals/stimuli”, “seed dormancy” and “seed maturation” were highly enriched, whereas within the down-regulated genes, genes related to “sugar and sulfur metabolic processes” were enriched (**Figure 10C**). Interestingly, DOG1-like 1 (At4g18660) and DOG1-like 3 (At4g18690) genes are up-regulated in *hd2ahd2b* seedlings, further confirming the repressive function of HD2A and HD2B on DOG1 gene family. Furthermore, transcription levels of genes responding to ABA and GA were also affected. These results demonstrate that HD2A and HD2B function is required for shutting down stimuli responses and genes involved in seed development and germination processes in ten days old seedlings.

In 24h imbibed seed, more genes are down-regulated (~1770) in *hd2ahd2b* than up-regulated (~520) (**Supplemental Figure S3**; **Supplemental Table S3**). GO term enrichment analysis revealed that, HD2A and HD2B are also involved in regulation of genes related to “response to stress and stimulus”. Moreover, the function of both plant-specific histone deacetylases is required for regulation of genes involved in “response to ABA”, “seed development”, “post-embryonic development”, and other developmental processes (**Supplemental Figure S3**). These results further confirm, that HD2A and HD2B function is required for regulating seed germination and further seedling development.

## Discussion

The plant-specific histone deacetylase subfamily HD2s plays multiple functions during plant development by acting as a transcription repressor (Wu et al., 2000; Colville et al., 2011). In this study, we showed that HD2A and HD2B may be recruited by HSI2 and HSL1 to the *DOG1* locus to regulate seed development and germination by repressing *DOG1*.

### HD2A and HD2B regulate *DOG1* expression

A previous study showed that HD2A and HD2C have contrasting roles in seed germination through glucose signaling, where HD2A restrains germination and HD2C promotes germination (Colville et al., 2011). In another study, HD2A function positively correlated with seed germination and negatively with dormancy-associated genes (Footitt et al., 2015). Similar to the later report, our results provide evidence that HD2A positively affects seed germination since *HD2A* single ko-mutant has an elevated *DOG1* expression level (**Figure 5A**). Surprisingly, we did not observe a significant difference in the seed germination phenotype between *hd2a* and WT (**Figure 1D**). Probably two-fold upregulation of *DOG1* in *hd2a* is insufficient for a detectable delay of germination. In contrast, *HD2B* knock-down resulted in a seven-fold upregulation of *DOG1* and significantly enhanced dormancy (**Figures 1D**, **5A**), which is consistent with previous reports (Yano et al., 2013). Both HD2A and HD2B are essential and functionally redundant for that process since the *hd2ahd2b* line has a significantly higher *DOG1* expression level (25-fold) and stronger dormancy phenotype in comparison to the corresponding single mutants (**Figures 1D**, **5A**). Although both, HD2A and HD2B functions, are important for controlling seed dormancy, the HD2B function seems to be more dominant in this process in comparison to HD2A. This is supported by a stronger effect of *HD2B* knock-down on germination and *DOG1* expression than the knock-out of *HD2A* (**Figures 1C, D**, **2**).

*DOG1* is a major genetic factor with a conserved function in controlling seed dormancy (Graeber et al., 2014; Huo et al., 2016). In mature and viable seeds, a higher *DOG1* transcript level is associated with stronger dormancy (Bentsink et al., 2006; Nakabayashi et al., 2012). Inter-accession variation of *DOG1* expression reflects the dormancy level of seeds of the different accessions. For example, Arabidopsis’s highly dormant accession Cvi has a higher *DOG1* expression level than the low-dormant accession Ler (Bentsink et al., 2006). In the highly dormant accession Cvi, the *DOG1* expression level is upregulated during seed development and peaked at 16 DAP, and decreased until seed maturation (Nakabayashi et al., 2012). In contrast, in low-dormant accession Col, the *DOG1* expression level peaked at 9 DAP and decreased until seed maturation (Zhao et al., 2015), indicating a different regulation mechanism of DOG1 in different dormant accessions. Our result confirmed the earlier decline of *DOG1* expression in the low-dormant accession Col (**Figure 5B**). The different *DOG1* expression levels might be a consequence of different *HD2B* expression levels since HD2B directly represses *DOG1* by deacetylating the *DOG1* coding region (**Figure 7**). At least, a natural variation of the *HD2B* expression is described for different Arabidopsis accessions. Arabidopsis highly dormant accession Cvi has a 25-fold lower *HD2B* expression level in comparison to the low-dormant accession Col (Yano et al., 2013).

We showed that HD2A and HD2B repress *DOG1* (**Figure 5A**). The higher *HD2B* expression during seed maturation contributes to the earlier decline of *DOG1* expression in low-dormant accessions. The earlier decline of *DOG1* expression, in turn, leads to less DOG1 accumulation in dry seeds and, subsequently, leads to a low-dormant phenotype, such as that of Col. In the *hd2ahd2b* line, without the repressing function of HD2A and HD2B, *DOG1* expression is continuously upregulated reaching the highest expression level in dry seeds (**Figure 5B**). Although *DOG1* expression dramatically decreased after imbibition in both, WT and *hd2ahd2b*, *hd2ahd2b* seeds still display a significantly higher *DOG1* expression than WT (**Figure 5B**). Interestingly, the *DOG1* expression level was dramatically increased in fresh dry seeds both in Col (**Figure 5C**) and Cvi accession (Bentsink et al., 2006). The precise mechanism behind that is still unknown. Probably unknown regulators with unique and independent functions from HD2A and HD2B accumulated during that developmental stage or *DOG1* mRNA stability is affected.

### HD2A and HD2B are interfering/interacting with ABA and GA signaling pathways

High *DOG1* expression level results in deeper seed dormancy. It was reported that the DOG1 protein level positively correlates with the ABA level in freshly harvested dry and imbibed seeds and negatively correlates with GA biosynthesis during imbibition (Nakabayashi et al., 2012). In this context, enhanced expression of *DOG1* in *hd2ahd2b* (**Figures 5A, B**) would indicate a higher ABA content and lower GA content. Surprisingly, we observed comparable amounts of ABA and GA3 in dry and imbibed seeds of WT and *hd2ahd2b* (**Figures 3C, D**) concluding that higher *DOG1* expression level in *hd2ahd2b* does not affect ABA and GA3 levels. This is further supported by the unchanged expression of ABA and GA signal transduction-related genes (**Figures 3A, B**). Interestingly, key regulatory genes of ABA and GA biosynthesis and ABA catabolism were upregulated in *hd2ahd2b* (**Figures 3A, B**), suggesting that HD2A and HD2B may be involved in regulating histone acetylation of these genes.

### HD2A and HD2B were recruited by HSL1 and HSI2 to mediate deacetylation of H4K5 at *DOG1*

Reduced expression of *HD2A* and *HD2B* resulted in a decrease in total HDA activity (**Figure 6A**), which in turn, led to increased global acetylation of histone H4 and H4K5 (**Figures 6B, C**). However, plant-specific HDAs themselves do most like not possess HDA activity but are rather required for the activity of RPD3-like HDAs. Previous studies (Luo et al., 2012b; Chen et al., 2018) provided evidence that HDAs are acting in multiple protein complexes. HD2A and HD2B seem to be two key subunits of such an HDA complex. Therefore, the loss of HD2A and HD2B function indirectly reduced HDA activity by disturbing the HDA protein complex. *DOG1* expression negatively correlated with the expression of *HD2A* and *HD2B* (**Figure 5A**). Moreover, the *hd2ahd2b* line has a significantly higher *DOG1* expression level and a stronger seed dormancy phenotype than the corresponding single mutants pointing to an overlapping function of HD2A and HD2B (**Figures 1C, D**, **5A, B**, **S1**). Loss of *DOG1* function in *hd2ahd2b* genetic background rescued the seed dormancy phenotype (**Figure 5C**), indicating that the upregulation of *DOG1* is the reason for the seed dormancy phenotype in *hd2ahd2b*. HD2B binds to the first exon of *DOG1* and deacetylates H4K5 and probably other acetylation marks of H4, too (**Figures 6B, C**, **7B, C**). The distance of around 200 - 500 bp downstream of the transcription start (TSS) is typically the regulatory region, where HDAs act. E. g. in S-nitrosoglutathione-treated Arabidopsis seedlings hyperacetylation of H3K9/14 was observed predominantly around 400 bp downstream of TSS (Mengel et al., 2017). Moreover, in *hda6* Arabidopsis mutant hyperacetylation of DNA peaked at 200 – 300 bp downstream of TSS (Ageeva-Kieferle et al., 2021).

HD2A and HD2B are interacting with each other (**Figure 8A**) and both plant-specific HDAs may function on *DOG1* binding sites. HDAs can be recruited to different DNA binding sites at different developmental stages and in different cell types *via* different complex partners or DNA binding proteins, such as transcription factors (Liu et al., 2014). Using a BIFC and Co-IP approach, we demonstrated that HD2A and HD2B interact with the transcriptional repressors HSI2 and HSL1 (**Figures 8B, C**), suggesting that during seed development and imbibition, HD2A and HD2B may be recruited by transcriptional repressors HSI2 and HSL1 to *DOG1*. This results in H4K5 deacetylation of *DOG1* and consequently in a decrease in the accessibility of *DOG1* for the transcription machinery. It was reported that the selectivity of the HDA activity largely depends on additional modifications of the substrate as well as corepressor binding (Riester et al., 2007; Liu et al., 2014). In yeast, the methyltransferase activity of DOT1 can be specifically activated by H4K16ac sites and is further enhanced by H2B ubiquitination (Valencia-Sánchez et al., 2021). Besides interacting with HD2A and HD2B, HSI2 and HSL1 also recruit CLF and LHP1 for consequent deposition of H3K27me3 marks at DOG1 to inhibit *DOG1* expression (Chen et al., 2020). In sum, repression of *DOG1* by HSI2 and HSL1 includes a combinatorial regulation *via* histone acetylation and methylation.

### Regulatory function of HD2A and HD2B in seed development and seed dormancy

Generally, a fully developed embryo, proper seed storage regents, and well seed coat characteristics (impermeable to water and/or oxygen and low mechanical resistance) are essential for seed dormancy and germination (Focks and Benning, 1998; Wang et al., 2016; Debeaujon et al., 2018). DOG1 plays a central role in regulating seed germination and is also involved in multiple aspects of seed maturation by interfering with ABA signaling components ABI3 and ABI5 (Dekkers et al., 2016).

We demonstrated that HD2A- and HD2B-mediated repression of *DOG1* is essential during seed development, maturation, and storage (**Figure 5B**), and loss of HD2A and HD2B function caused multiple defects in seeds (**Figures 9A–H**). Our transcriptomic data provided a general insight into the functions of HD2A and HD2B. GO enrichment analysis demonstrated that these genes are involved in seed maturation, seed dormancy, and seed development process (**Figure 10C**; **Supplemental Figure S3**). It was reported, that DOG1 mediates a conserved coat dormancy mechanism that controls seed germination through the regulation of GA metabolism (Graeber et al., 2014). In this context, it is important to note that the expression of genes involved in “GA biosynthetic processes” and “response to GA” is disturbed in *hd2ahd2b* (**Figure 10C**). Interestingly, HD2A and HD2B function is also required to control the expression of genes responding to different types of stimuli (**Figure 10C**; **Supplemental Figure S3**). Coordinated responses to external or environmental stimuli are important to cope with environmental changes.

**Figure 11:**
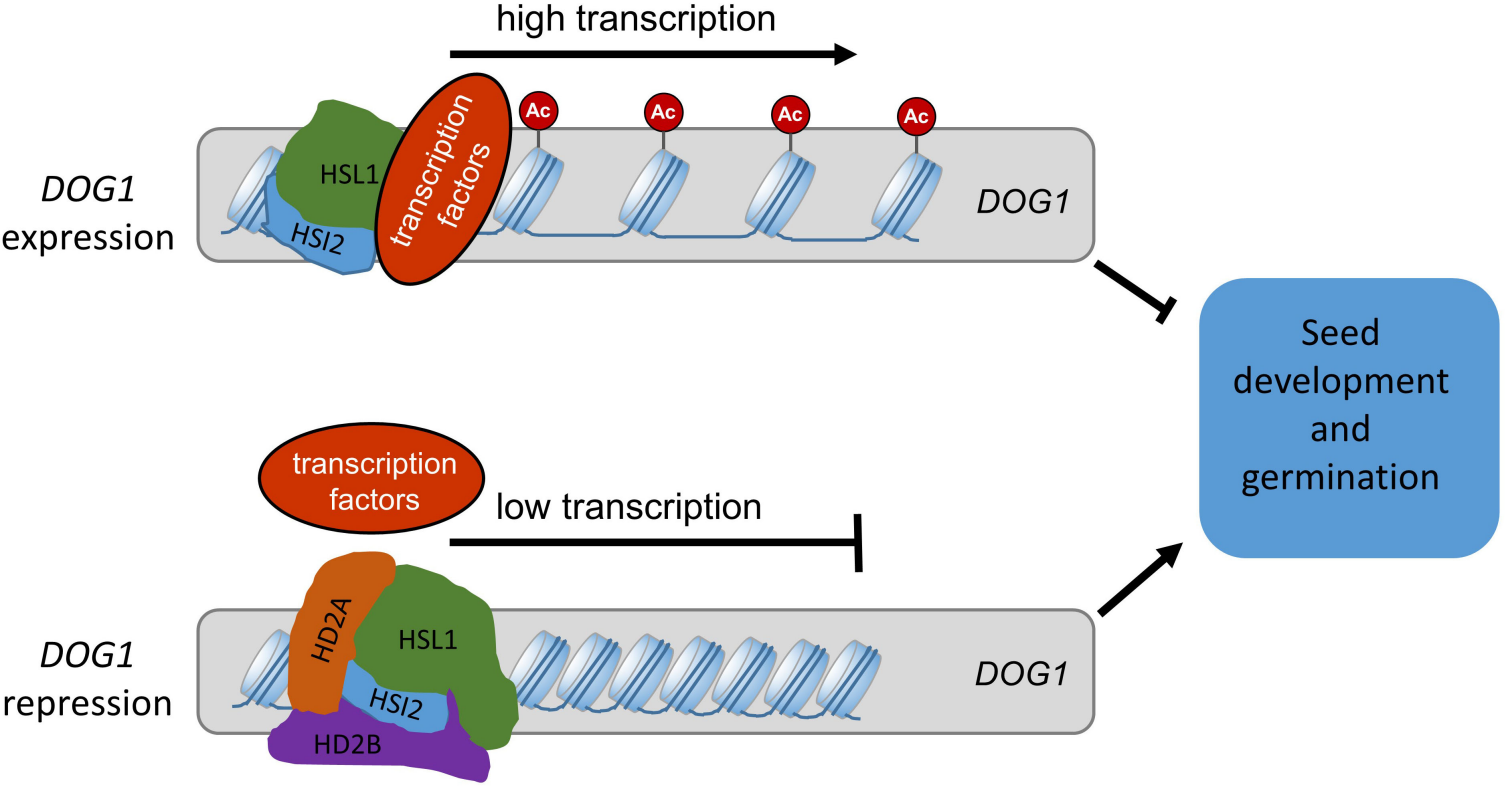
Proposed model for the regulation of seed dormancy and germination mediated by HD2A and HD2B. H4ac and H4K5ac at *DOG1* result in open chromatin structure and enable *DOG1* transcription. The hetero-oligomer of HD2A and HD2B is recruited by HSI2 and HSL1 and directly binds to the coding region of *DOG1*, causing a decrease in H4ac and H4K5ac levels. Consequently, the *DOG1* expression level is reduced during seed maturation and imbibition. The gradually increased expression of *HD2A* and *HD2B* guarantees normal seed development during seed maturation and release of the seed dormancy during seed imbibition. AC, acetyl groups.

In sum, we showed, that the Arabidopsis plant-specific histone deacetylases HD2A and HD2B have a redundant function and are involved in controlling seed development and germination by coordinating *DOG1* expression. Based on our results, we propose a model for the regulatory function of HD2A and HD2B in seed development/germination processes (**Figure 11**). Acetylation of H4K5 at the 5´-end of the coding region of *DOG1* enables its transcription and establishes seed dormancy. During seed maturation and imbibition, HSI2 and HSL1 may recruit HD2A and HD2B to the 5´-end of the coding region of *DOG1*. Consequently, this region is deacetylated at H4K5 and resulting in the repression of *DOG1*.

## Data availability statement

The RNA sequencing data presented in the study are deposited in the ENA repository (https://www.ebi.ac.uk/ena/), accession number PRJEB62044.

## Author contributions

YH and CL conceived research plans; CL, CB, GH, and RK supervised the experiments; YH, SPC, CW, and PH performed the experiments; YH, CL, EG, SPC, GH, and RK designed the experiments and analyzed the data; YH and CL wrote the article with contributions of all the authors; EG, CB, and JD reviewed and edited the text. CL agrees to serve as the author responsible for contact and ensures communication. All authors contributed to the article and approved the submitted version.

## Supplementary material

Supplementary Figure 1 Germination analysis of different *hd2* lines.

Supplementary Figure 2 Germination of freshly harvested wild-type and HD2s mutant seeds.

Supplementary Figure 3 HD2A and HD2B function is required for the regulation of genes involved in different developmental processes and ABA signaling in 24h imbibed seeds.

Supplementary Table 1 List of oligonucleotides.

Supplementary Table 2 RNA-seq data set_control_ab vs wt (seedlings).

Supplementary Table 3 RNA-seq data set_ab vs wt (24h imbibed seeds).
