# Supplementary material for: Arabidopsis histone deacetylase HD2A and HD2B regulate seed dormancy by repressing DELAY OF GERMINATION 1
Source: Front Plant Sci. 2023 May 29;14:1124899. doi: 10.3389/fpls.2023.1124899 (PMC10258333; doi:10.3389/fpls.2023.1124899)
Supplement: Supplementary file 1 [file DataSheet_1.pdf]

Supplementary Information: Methods

Arabidopsis Histone Deacetylase HD2A and HD2B Regulate Seed Dormancy by Repressing DELAY OF GERMINATION 1

Seed germination assays

For "fresh seed germination tests", seeds were harvested as soon as the siliques became dehiscent and were used immediately for germination assays. For "after ripening germination tests", the seeds were harvested and stored dry at room temperature in the dark until used for the germination test. For germination tests, the seeds were sown on water-saturated filter paper without any additional treatment and germination rates were analysed after 3 days of incubation under long-day conditions in the growth chamber. For the seed germination assay under different ABA concentration, the 16 weeks after-ripened seeds were sterilized with 75% (v/v) ethanol for 1min in the 1.5mL micro tube and air dried on a sterilized filter paper before sown on half-strength Murashige and Skoog (MS) medium without sucrose but with different concentration ABA. The 100mM ABA stock solution was prepared by dissolving ABA powder in pure ethanol and diluted to the working concentration in MS medium. For the seed germination assay under GA3 treatment, the GA3 was dissolved in water to reach the final concentration of 100 μM. The fresh and 16 weeks after-ripened seeds were planted on the GA3 solution-saturated filter paper. The planted seeds were directly incubated under long-day condition growth chamber without any additional treatment. For each independent experiment, approximately 100 freshly harvested or after ripened seeds were sown directly onto a ½ MS plate or water-saturated filter paper. Germination ratio was scored as "radicle emergence".

Constructs and plant transformation

To generate *35Spro:HD2A-GFP* and *35Spro:HD2B-GFP* constructs, the open reading frame (ORF) of *HD2A* and *HD2B* was PCR amplified from cDNA using iProof polymerase (Biorad, catalog no. 1725301), cloned into pDONR221 (Invitrogen) and sequenced. Subsequently, the *pDONR221-HD2A* and *pDONR221-HD2B* constructs were recombined by Multisite Gateway Cloning into the binary destination vector pK7WGF2,0 containing a GFP open reading frame. To build the HD2A-nYFP, HD2B-nYFP, HD2B-cYFP, HSI2-cYFP, and HSL1-cYFP constructs, the corresponding ORFs were cloned into the pBiFCt-2in1-NN vector and the 2 in 1 vector was constructed according to the described (Grefen & Blatt, 2012). For *35Spro:HSI2-myc* and *35Spro:HSL1-myc* constructs, the ORF of Arabidopsis HSI2 and HSL1 was cloned,

fused N-terminal to a MYC-tag and recombined into the binary destination vector pCAMBIA 2300 by homologous recombination. All used primers are listed in **Supplementary Table 1**. The *35Spro:HD2A-GFP* and *35Spro:HD2B-GFP* constructs were transferred into *Agrobacterium tumefaciens* GV3101 by electroporation and for transformation of Arabidopsis, the plants were infected by the floral dipping method(Clough & Bent, 1998). T3 homozygous lines were used for further analysis. Arabidopsis Genomic DNA has been extracted from 10 days seedlings with a modified CTAB method and 100 ng gDNA was used as a template for genotyping the transformed plants and T-DNA insertion lines.

DNA extraction, RNA extraction, cDNA synthesis, and gene expression analyses

Total RNA was extracted from developing Arabidopsis siliques, dry seeds or imbibed seeds using Trizol reagent (Meng & Feldman, 2010) and purified with RNeasy Plant Mini Kit (Qiagen, catalog no. 74904) according to the manufacturer's instruction. 0.5 µg of total purified RNA was used for the cDNA synthesis with random hexamer primers using a QuantiTect Rev. Transcription Kit (Qiagen, catalog no. 205311). The cDNA was diluted 50-fold with water for subsequent PCR experiments. RT-qPCR was performed in a 20 µl reaction with 10 µl Sybr green (Bioline, catalog no. QT625-05), 1 µl of 10 µM specific primers, 5 µl of dH2O, and 3 µl of diluted cDNA. The PCR conditions were as follows: 10 min at 95°C, followed by 45 cycles of 15 s at 95°C, 15 s at 55°C, and 45 s at 72°C, and a dissociation step, 15 s at 95°C, 60 s at 60°C, and 15 s at 95°C. RT–qPCR results were normalized using *UBQ5* as an internal control. The primers used for RT-qPCR are listed in **Supplementary Table 1**. At least three biological replicates were analysed. The relative expression levels of target genes were calculated with formula $2^{-\Delta\Delta CT}$.

RNA-seq analysis

Arabidopsis WT and *hd2ahd2b* plants were grown under short-day conditions for 10 days. Total RNA of four biological replicates was extracted with RNeasy Plant Mini Kit (Qiagen, catalog no. 74904) according to the manufacturer's instructions. Single-end sequencing of all RNA samples was performed using two lanes of a NovaSeq SP system (Illumina). Reads were aligned against the TAIR10 genome assembly (Berardini, 2015) by HISAT2-2.1.0 (Kim *et al.*, 2015). The alignments were sorted using Samtools-1.8 (Li *et al.*, 2009) and expression quantification was done with Stringtie-1.3.4 (Pertea *et al.*, 2015) for the Araport11 genome annotation (Berardini *et al.*, 2015). Differential expression was computed via the R package DESeq2,

version 1.20.0 (Love *et al.*, 2014). GO enrichment and multi-dimensional scaling analyses were performed in R (Ageeva-Kieferle *et al.*, 2021).

Protein extraction and immunostaining

The histone proteins were extracted from the nucleus by acidic extraction. The harvested plant material (approximately 0.5 g germinated seeds) were ground in liquid nitrogen and the powder was re-suspended on ice in 5 ml pre-cooled histone extraction buffer (0.3 M Sucrose, 40 mM $NaHSO_3$, 25 mM Tris pH 7.4, 10 mM $MgSO_4$, 0.5 mM EDTA, 0.5% NP40, 1 mM PMSF, 1x Protease Inhibitor). After filtering through Mira cloth, the filtrate was centrifuged at 10,000*g* for 10 min. The pellet was re-suspended and washed two times with cold histone extraction buffer. The nuclei were extracted twice with 400 µl of 0.4 M H_2SO_4 and incubated overnight on a rotator at 4°C to dissolve the histones. The sample was centrifugated at 12000g and 4°C for 10 min, and the supernatant was transfer into a new tube and mix with 200 µl of 100% TCA. After incubation on ice for 1 h, the sample was centrifugated at 12000g for 10 min and the supernatant was discarded. The pellet was washed two times in ice-cold acetone at room temperature and dried. Histones were dissolved in 20 mM Tris pH 8.0 and their concentration was determined with bradford reagent (Biorad, catalog no. 5000006) (Zhang *et al.*, 2019). Five µg of histone proteins were used for western-blot analysis following standard protocols. Briefly, the proteins were separated on 10% SDS-PAGE gel and then transferred to a PVDF membrane (Abcam) using a semi-dry western blot system. The membrane was blocked in 5 % BSA/TBS-T (10 mM Tris/HCl pH 7.5, 0.9 % NaCl, 1 mM MgCl2, 0.05 % Tween 20) blocking buffer for 2 h at room temperature, followed by incubation with the primary antibody in blocking buffer overnight at 4 °C. The following primary antibodies and dilutions were used: anti-H3 (1:500; Millipore; catalog no. 05499), anti-H4 (1:1000; Abcam; catalog no. 31830), anti-acetylated H4 (1:20000; Millipore; catalog no. 06866), anti-acetylated H3K9 (1:5000; Abcam; catalog no. 10812), and anti-acetylated H4K5 (1:10000; Abcam). Afterward, the membrane was washed four times for 10 min with 1 x TBS-T buffer and incubated for 1 h at room temperature with secondary antibody, anti-rabbit IgG (1:2500; catalog no. W54011) or anti-mouse IgG (1: 1000; Agrisera; catalog no. AS122472) linked to horseradish peroxidase in blocking buffer. Afterwards, the membrane was washed with 20 ml of 1x TBS-T buffer and two times with 20 ml of 1x TBS buffer. Bound antibodies were detected using western lightning plus-ECL chemiluminescence substrate (PerkinElmer, Cat No NEL105001EA). Four replications were performed and the intensities of the signals of the immunoblot were quantified using the ImageJ software.

Protoplasts isolation and transient expression

Arabidopsis protoplasts were generated as described previously (Yoo *et al.*, 2007; Wu *et al.*, 2009). About 40 abaxial epidermis of 3 weeks old rosette leaves was removed and cell walls were digested in 20 ml of enzyme solution (0.4 M mannitol, 20 mM KCl, 20 mM MES, pH 5.7, 10 mM $MgCl_2$, 0.1% BSA, 1.5% cellulase, and 0.8% macerozyme) for 3 h at room temperature (dark, slightly shaking). The enzyme/protoplast suspension was diluted with 20 ml of W5 solution (154 mM NaCl, 2 mM MES, pH 5.7, 125 mM CaCl*2*, and 5mM KCl) and the protoplast suspension was filtered through 75-μm nylon mesh. Protoplasts were washed two times with 20 ml of cold W5 (100*g*, 2 min, 4°C), and re-suspended in ice-cold W5 solution. The concentration of the protoplasts was determined by counting under the microscope and was adjusted to 10^6 cells ml^{-1}. Protoplasts were transfected as describe (Yoo *et al.*, 2007). 10 μl plasmid (1 μg/μl) was mixed with 100 μl of protoplast in MMG solution (4 mM MES (pH 5.7), 0.4 M mannitol and 15mM $MgCl_2$) and 110 μl freshly prepared PEG solution (40% PEG 4000, 0.2 M mannitol and 100 mM $CaCl_2$) was added to the protoplast suspension and mix gently. After 10 min incubation at room temperature, the sample was diluted with 440 μl of W5 solution and mixed gently. The transfected protoplasts were washed two times with W5 solution and transferred into a cell culture dish which was prewashed with 10% glycerine, and incubated at room temperature for 24 h before fluorescence detection.

References

Ageeva-Kieferle A, Georgii E, Winkler B, Ghirardo A, Albert A, Hüther P, Mengel A, Becker C, Schnitzler J-P, Durner J, et al. 2021. Nitric oxide coordinates growth, development, and stress response via histone modification and gene expression. *Plant Physiology*.

Berardini TZ, Reiser L, Li D, Mezheritsky Y, Muller R, Strait E, Huala E. 2015. The Arabidopsis information resource: Making and mining the "gold standard" annotated reference plant genome. *Genesis* **53**(8): 474-485.

Berardini TZ, Reiser, L., Li, D., Mezheritsky, Y., Muller, R., Strait, E., Huala, E. 2015. The Arabidopsis information resource: Making and mining the "gold standard" annotated reference plant genome. *Genesis* **53**(8): 474-485.

Clough SJ, Bent AF. 1998. Floral dip: a simplified method for Agrobacterium-mediated transformation of Arabidopsis thaliana. *Plant Journal* **16**(6): 735-743.

Grefen C, Blatt MR. 2012. A 2in1 cloning system enables ratiometric bimolecular fluorescence complementation (rBiFC). *Biotechniques* **53**(5): 311-314.

Kim D, Langmead B, Salzberg SL. 2015. HISAT: a fast spliced aligner with low memory requirements. *Nat Methods* **12**(4): 357-360.

Li H, Handsaker B, Wysoker A, Fennell T, Ruan J, Homer N, Marth G, Abecasis G, Durbin R. 2009. The Sequence Alignment/Map format and SAMtools. *Bioinformatics* **25**(16): 2078-2079.

Love MI, Huber W, Anders S. 2014. Moderated estimation of fold change and dispersion for RNA-seq data with DESeq2. *Genome Biol* **15**(12): 550.

Meng L, Feldman L. 2010. A rapid TRIzol-based two-step method for DNA-free RNA extraction from Arabidopsis siliques and dry seeds. *Biotechnology Journal* **5**: 183-186.

Pertea M, Pertea GM, Antonescu CM, Chang TC, Mendell JT, Salzberg SL. 2015. StringTie enables improved reconstruction of a transcriptome from RNA-seq reads. *Nat Biotechnol* **33**(3): 290-295.

Wu F-H, Shen S-C, Lee L-Y, Lee S-H, Chan M-T, Lin C-S. 2009. Tape-Arabidopsis Sandwich - a simpler Arabidopsis protoplast isolation method. *Plant Methods* **5**(1): 16.

Yoo SD, Cho YH, Sheen J. 2007. Arabidopsis mesophyll protoplasts: a versatile cell system for transient gene expression analysis. *Nature Protocol* **2**(7): 1565-1572.

Zhang J, Buegger F, Albert A, Ghirardo A, Winkler B, Schnitzler J-P, Hebelstrup KH, Durner J, Lindermayr C. 2019. Phytoglobin overexpression promotes barley growth in the presence of enhanced level of atmospheric nitric oxide. *Journal of Experimental Botany* **70**(17): 4521-4537.
