# Supplementary figures and images for: Arabidopsis histone deacetylase HD2A and HD2B regulate seed dormancy by repressing DELAY OF GERMINATION 1
Source: Front Plant Sci. 2023 May 29;14:1124899. doi: 10.3389/fpls.2023.1124899 (PMC10258333; doi:10.3389/fpls.2023.1124899)

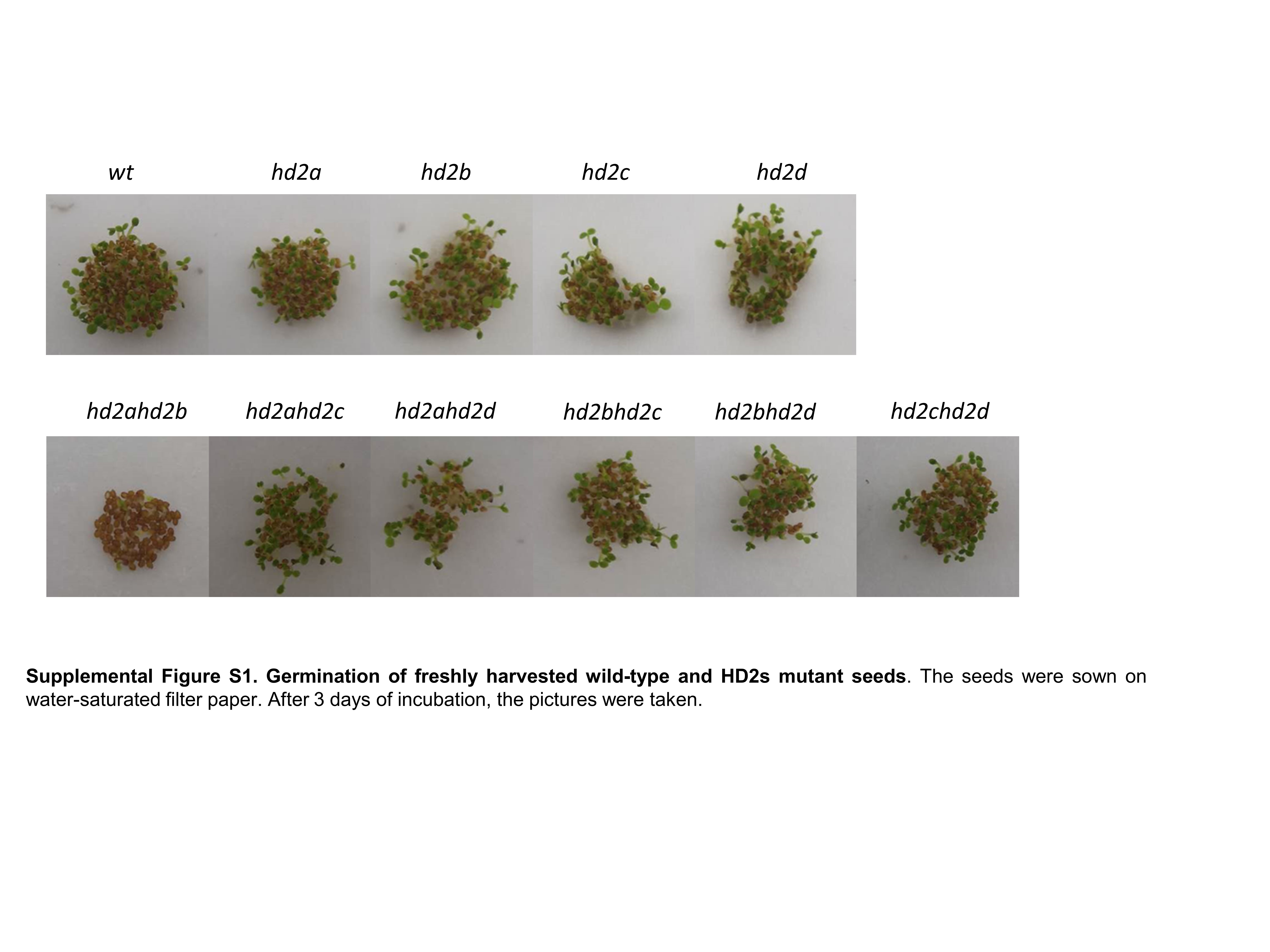

Supplement: Supplementary Figure 1 — Germination analysis of different hd2 lines. [file Image_1.jpeg]

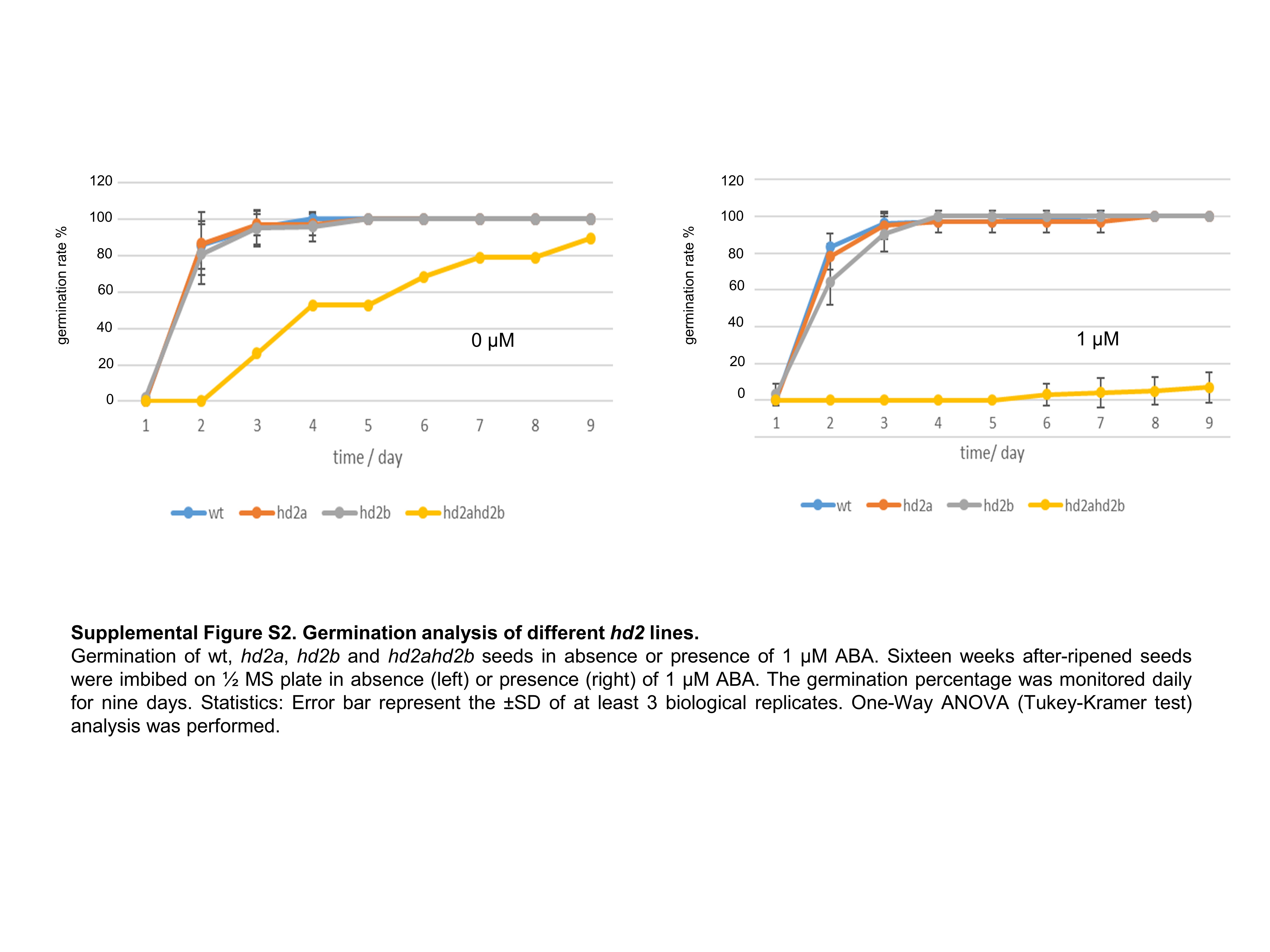

Supplement: Supplementary Figure 2 — Germination of freshly harvested wild-type and HD2s mutant seeds. [file Image_2.jpeg]

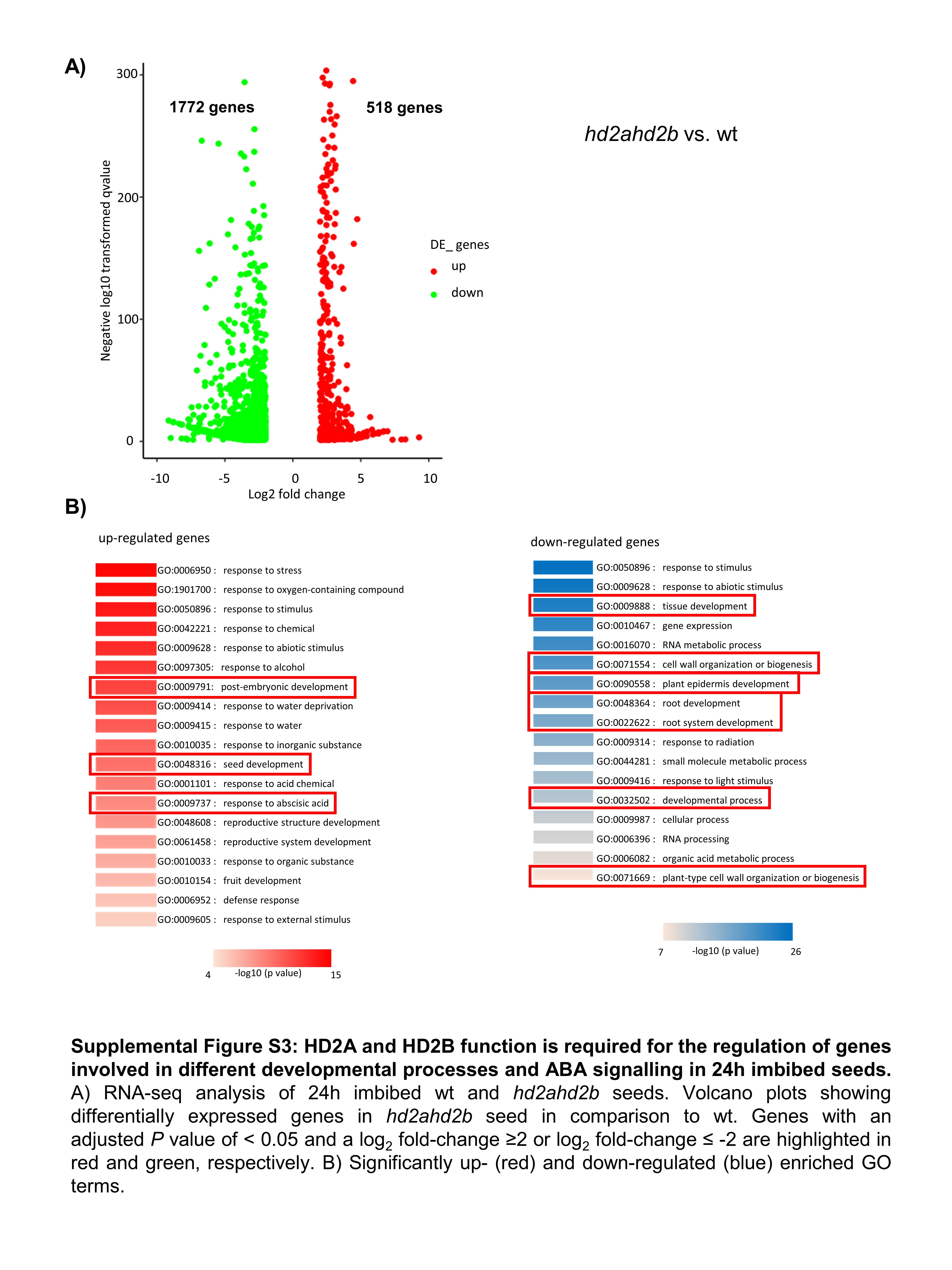

Supplement: Supplementary Figure 3 — HD2A and HD2B function is required for the regulation of genes involved in different developmental processes and ABA signaling in 24h imbibed seeds. [file Image_3.jpeg]
