# Supplementary material for: Arabidopsis histone deacetylase HD2A and HD2B regulate seed dormancy by repressing DELAY OF GERMINATION 1
Source: Front Plant Sci. 2023 May 29;14:1124899. doi: 10.3389/fpls.2023.1124899 (PMC10258333; doi:10.3389/fpls.2023.1124899)
Supplement: Supplementary Table 1 — List of oligonucleotides. [file Table_1.pdf]

List of oligonucleotides

Name	Nucleotide sequence	Used for
HD2A-LP	CGGCTTCGTATTAAAACCCTC	Genotyping HD2A
HD2A-RP	GCCTTTGGTTTAGCTACAGCC	Genotyping HD2A
HD2B-LP	TCTTCTTCTCCTCCCGATAGC	Genotyping HD2B
HD2B-RP	AACAAGTTTAGCCCCACCAAC	Genotyping HD2B
HD2C-LP	ATCCAGTCCTGCAATGATTTG	Genotyping HD2C
HD2C-RP	TTCTTCTTCTCAAGCAGCTGC	Genotyping HD2C
HD2D-LP	TCTCATTTTGAGGCAAAGCAG	Genotyping HD2D
HD2D-RP	CAAAAGCTTGCCTTTCTTCTG	Genotyping HD2D
attb1-GFP-HD2A	GGGGACAAGTTTGTACAAAAAAGCAGGCTTAATGGAGTTCTGGGGAATTG	HD2A OX line
attb2-GFP-HD2A	GGGGACCACTTTGTACAAGAAAGCTGGGTCTCACTTGGCAGCAGCGTGCT	HD2A OX line
attb1-GFP-HD2B	GGGGACAAGTTTGTACAAAAAAGCAGGCTTAATGGAGTTCTGGGGAGTTG	HD2B OX line
attb2-GFP-HD2B	GGGGACCACTTTGTACAAGAAAGCTGGGTCTTAAGCTCTACCCTTTCCCTTG	HD2B OX line
nYFP-HD2A-attb3	GGGGACAACTTTGTATAATAAAGTTGTAATGGAGTTCTGGGGAATTGAAG	BIFC
nYFP-HD2A-attb2	GGGGACCACTTTGTACAAGAAAGCTGGGTCTCACTTGGCAGCAGCGTGCT	BIFC
nYFP-HD2B-attb3	GGGGACAACTTTGTATAATAAAGTTGTAATGGAGTTCTGGGGAGTTGCG	BIFC
nYFP-HD2B-attb2	GGGGACCACTTTGTACAAGAAAGCTGGGTCTTAAGCTCTACCCTTTCCCTTG	BIFC
cYFP-HD2A-attb1	GGGGACAAGTTTGTACAAAAAAGCAGGCTTAATGGAGTTCTGGGGAATTG	BIFC
cYFP-HD2A-attb4	GGGGACAACTTTGTATAGAAAAGTTGGGTGCTTGGCAGCAGCGTGCTT	BIFC
cYFP-HSI2-attb1	GGGGACAAGTTTGTACAAAAAAGCAGGCTTAATGTTTGAAGTCAAAATGGGG	BIFC
cYFP-HSI2-attb4	GGGGACAACTTTGTATAGAAAAGTTGGGTGTCAGCTTGAAACTCTCGGCTCTT	BIFC
cYFP-HSL1-attb1	GGGGACAAGTTTGTACAAAAAAGCAGGCTTAATGGAGTCAATAAAGGTTTGCA	BIFC
cYFP-HSL1-attb4	GGGGACAACTTTGTATAGAAAAGTTGGGTGTTAGTTCACAGGATCATGAGCTC	BIFC
myc-HSI2-2300-F	gagctcggtacccggggatccGAGCAGAAACTCATCTCTGAAGAGGATCTGATGTTTG AAGTCAAAATGGGG	co-ip
myc-HSI2-2300-R	caccatggtgtcgactctagaTCAGCTTGAAACTCTCGGCTCTT	co-ip
myc-HSL1-2300-F	gagctcggtacccggggatccGAGCAGAAACTCATCTCTGAAGAGGATCTGATGGA GTCAATAAAGGTTTGCA	co-ip
myc-HSL1-2300-R	caccatggtgtcgactctagaTTAGTTCACAGGATCATGAGCTC	co-ip

negative control-CHIP QPCR-F	CAGTCTCTGCGAAGGGGATC	Chip-qPCR
negative control-CHIP QPCR-R	GCTTCACAAGACACGGTTCG	Chip-qPCR
DOG1-chip-P1-F	CTACACCTCTTCTTAAGTCTCTGATC	Chip-qPCR
DOG1-chip-P1-R	GGATGTCCTGAGACAGCGGATTG	Chip-qPCR
DOG1-chip-P2-F	TGCAATCTCAACGCATCCCT	Chip-qPCR
DOG1-chip-P2-R	TGTTCCACGTGGGTGCATAA	Chip-qPCR
GA3OX1-F	CCACCGGTGAAAACATCCCT	qPCR
GA3OX1-R	CGCCGTGGTTTGAGATTTGG	qPCR
GA3OX2-F	AGTTTTCCGGGATGACGTGG	qPCR
GA3OX2-R	CTCGGGAAGATTCCGTTGGT	qPCR
GA2OX2-F	CCCTCAAATTTTCCGTGAGT	qPCR
GA2OX2-R	CAGCATTTTACTCAGAGTGTC	qPCR
GID1b-F	CTTCCATGGAGGCAGCTTCA	qPCR
GID1b-R	TGACCCAGTTGAGAGCGTTC	qPCR
GID1c-F	TTGGAGGGACCGAAAGAACG	qPCR
GID1c-R	TCACCCTCAGGAAGAAACGC	qPCR
wri1-F-qpcr	CACCACAACGGAAGATGGGA	qPCR
wri1-R-qpcr	ACTCAATCGCAGCCATGTCA	qPCR
ABI5-F	GAGAATGCGCAGCTAAAACA	qPCR
ABI5-R	GTGGACAACTCGGGTTCCTC	qPCR
ABA1-F	GGCATTTGGTCTAAGGTGAGAA	qPCR
ABA1-R	CAGACTCGATATCCGCTGGTA	qPCR
CYP707A2-F	ATCCTCCAAGCAAAGAGCGT	qPCR
CYP707A2-R	GAGCTCGATGTGAGAGACGG	qPCR
NCED3-F	CCGGAGAAGGAGGAGAGGAA	qPCR
NCED3-R	CTGCTTCGCCAAATCATCGG	qPCR
ABI2-F	GTGGTTGCGGTTGTCTTTCC	qPCR
ABI2-R	GGATTACTTTCCCACCGGCA	qPCR
SnrK2.3-F	GATGGAAGTCCTGCTCCTCG	qPCR

SnrK2.3-R	GAAGCAGTACCTCTGGAGCG	qPCR
HD2A-F	TGAGCCACAAGGCTATTCTGAGG	qPCR
HD2A-R	AGCTACAGCCTTGGCAGCATTC	qPCR
HD2B-F	TGTTCCTGAAGCTGTTCCTGCTC	qPCR
HD2B-R	AACAGCTGCTCCAGCATTTCCG	qPCR
HD2C-F	ACGACGAAGAGGATGACTCCTCAG	qPCR
HD2C-R	CTCTTCTTGGGTTCTTCAGGCTTC	qPCR
HD2D-F	TCTTCTCAAGCAGCCACATCTTCC	qPCR
HD2D-R	TCCCTGGCTTAATCTCGATACCC	qPCR
DOG1-F	AAAATTACGCCGCAAAAAGA	qPCR
DOG1-R	CCGCCACCACCTGAAGATTCGTAG	qPCR
UBQ5-F	GGTGCTAAGAAGAGGAGGAAT	qPCR
UBQ5-R	CTCCTTCTTCTGGTAAACGT	qPCR
S16-F	TTTACGCCATCCGTCAGAGTAT	qPCR
S16-R	TCTGGTAACGAGAACGAGCAC	qPCR
